# Treatment of a metabolic liver disease in mice with a transient prime editing approach

**DOI:** 10.1038/s41551-025-01399-4

**Published:** 2025-05-20

**Authors:** Tanja Rothgangl, András Tálas, Eleonora I. Ioannidi, Yanik Weber, Desirée Böck, Mai Matsushita, Elina Andrea Villiger, Lukas Schmidheini, Woohyun J. Moon, Paulo J. C. Lin, Steven H. Y. Fan, Kim F. Marquart, Cornelia Schwerdel, Nicole Rimann, Erica Faccin, Lukas Villiger, Hiromi Muramatsu, Máté Vadovics, Alessio Cremonesi, Péter István Kulcsár, Beat Thöny, Manfred Kopf, Johannes Häberle, Norbert Pardi, Ying K. Tam, Gerald Schwank

**Affiliations:** 1 Institute of Pharmacology and Toxicology, University of Zurich, Zurich, Switzerland; 2 Institute of Molecular Health Sciences, ETH Zurich, Zurich, Switzerland; 3 Acuitas Therapeutics Inc., Vancouver, British Columbia Canada; 4 Division of Metabolism and Children’s Research Center, University Children’s Hospital Zurich, Zurich, Switzerland; 5 Department of Microbiology, Perelman School of Medicine, University of Pennsylvania, Philadelphia, PA USA; 6 Division of Clinical Chemistry and Biochemistry, University Children’s Hospital Zurich, University of Zurich, Zurich, Switzerland

**Keywords:** Targeted gene repair, Genetic engineering

## Abstract

Prime editing is a versatile genome editing technology that circumvents the need for DNA double-strand break formation and homology-directed repair, making it particularly suitable for in vivo correction of pathogenic mutations. Here we developed liver-specific prime editing approaches with temporally restricted prime editor (PE) expression. We first established a dual-delivery approach where the prime editor guide RNA is continuously expressed from adeno-associated viral vectors and only the PE is transiently delivered as nucleoside-modified mRNA encapsulated in lipid nanoparticles (LNP). This strategy achieved 26.2% editing with PEmax and 47.4% editing with PE7 at the *Dnmt1* locus using a single 2 mg kg^−1^ dose of mRNA–LNP. When targeting the pathogenic *Pah*^*enu2*^ mutation in a phenylketonuria mouse model, gene correction rates reached 4.3% with PEmax and 20.7% with PE7 after three doses of 2 mg kg^−1^ mRNA–LNP, effectively reducing blood l-phenylalanine levels from over 1,500 µmol l^−1^ to below the therapeutic threshold of 360 µmol l^−1^. Encouraged by the high efficiency of PE7, we next explored a simplified approach where PE7 mRNA was co-delivered with synthetic prime editor guide RNAs encapsulated in LNP. This strategy yielded 35.9% editing after two doses of RNA–LNP at the *Dnmt1* locus and 8.0% editing after three doses of RNA–LNP at the *Pah*^*enu2*^ locus, again reducing l-phenylalanine levels below 360 µmol l^−1^. These findings highlight the therapeutic potential of mRNA–LNP-based prime editing for treating phenylketonuria and other genetic liver diseases, offering a scalable and efficient platform for future clinical translation.

## Abstract (web summary)

A transient in vivo prime editing strategy is developed for the liver by delivering the prime editor as mRNA encapsulated in LNP.

## Main

Phenylketonuria (PKU) is an autosomal recessive metabolic liver disease caused by mutations in the phenylalanine hydroxylase (*PAH*) gene. While untreated PKU causes severe retardation, microcephaly and seizures, newborn screening followed by dietary l-phenylalanine (Phe) restriction and enzyme therapy leads to a life expectancy comparable to healthy individuals^1–4^. Nevertheless, despite existing treatments, learning disabilities and attention deficits remain frequent in PKU patients. In addition, the intricate dietary guidelines place a substantial burden on the quality of life. As a result, new treatment strategies attempting to permanently restore PAH expression in the liver are under exploration. Despite classical gene addition therapies, which provide an additional functional *PAH* gene copy to hepatocytes^5^, there is a growing interest in genome editing technologies that aim to directly repair pathogenic variants. The main advantage of gene correction is the sustained expression of the corrected allele after the edit is installed, even as hepatocytes divide and replicate their genomes. Hence, in contrast to gene addition approaches that rely on DNA delivery for prolonged transgene expression, gene correction can also be achieved using transient methods, such as lipid nanoparticle (LNP)-mediated RNA delivery^6^.

Using the *Pah*^*enu2*^ mouse model for human PKU, which contains a point mutation (c.835T>C; p.F263S) that abolishes PAH function and results in an increase of Phe levels from 60 µmol l^−1^ to 1,500 µmol l^−1^ (ref. ^7,8^), Richards et al.^9^ explored the feasibility of correcting this metabolic liver disease using clustered regularly interspaced short palindromic repeats and CRISPR–Cas9 nucleases. However, to the low frequency of homology-directed repair (HDR) in the liver, only 1% of hepatocytes were corrected, and hyperphenylalaninaemia could not be resolved. Circumventing the need for HDR, we and others have previously used base editing to repair pathogenic PKU mutations at rates that led to therapeutic reduction of Phe levels^8,10–12^. Nonetheless, even though base editing holds promise for clinical use in PKU patients, the technology is largely limited to the correction of transition point mutations, excluding patients with other types of mutation.

Similar to base editing, prime editing allows precise correction of mutations without the need for HDR. Prime editors (PEs) consist of a H840A *Sp*Cas9 nickase (nCas9) fused to an engineered Moloney murine leukaemia virus (M-MLV) reverse transcriptase (hereafter referred to as PE2)^13^. This complex is guided to the locus of interest by the prime editor guide RNA (pegRNA), which contains the reverse transcriptase template (the RTT) and the primer binding site fused to the 3′ end of the guide RNA scaffold. nCas9-mediated nicking of the non-target DNA strand and hybridization of the primer binding site allows the reverse transcriptase to elongate the 3′ DNA end using the RTT sequence as a template. Successful incorporation of the generated 3′ flap into the locus results in the installation of the intended edit. Prime editing therefore enables the introduction of theoretically any small-sized genetic change. Several recent studies provide proof of concept for in vivo prime editing in the liver^11,14–18^. However, when using a transient prime editing approach where the PE and pegRNA were delivered via RNA–LNP, editing rates remained relatively low (below 8% at the Pcsk9 locus despite re-dosing)^19^. While higher editing rates were achieved in studies where the PE was delivered via viral vectors^11,17,20–22^, the resulting permanent expression of the PE could pose challenges for clinical applications, as it elevates the likelihood of installing unintended off-target mutations and potentially triggers T-cell-mediated elimination of edited cells that continuously express the genome editor.

In this study, we developed transient in vivo prime editing strategies for the liver by delivering the PE as mRNA encapsulated in LNP. Using adeno-associated virus (AAV) to express the pegRNAs, we achieved editing efficiencies of up to 47.4% at the *Dnmt1* locus and 20.7% at the *Pah*^*enu2*^ locus. When pegRNAs were co-delivered as synthetic, chemically modified RNAs, editing efficiencies reached up to 35.9% and 8.0% at the respective loci. Both delivery methods successfully reduced Phe levels to below the therapeutic threshold of 360 µmol l^−1^, demonstrating the potential for mRNA–LNP-mediated prime editing approaches in correcting genetic liver disorders.

## Results

### Prime editing of *Pah*^*enu2*^ mice using AAV delivery

In a previous study, we attempted to correct the pathogenic mutation in the *Pah*^*enu2*^ mouse model for PKU using AAV-mediated delivery of intein-split PE2 and a pegRNA targeting *Pah*^*enu2*^ (mPKU-2.1)^17^. However, despite applying AAV doses of 1 × 10^14^ vector genomes (vg) per kg, correction rates remained below 1%, and treatment was only successful when prime editing components were delivered from adenoviral vectors at doses that are not viable in a clinical context. This prompted us to test whether recent improvements in the prime editing technology could increase correction rates of the *Pah*^*enu2*^ mutation.

**Fig. 1:**
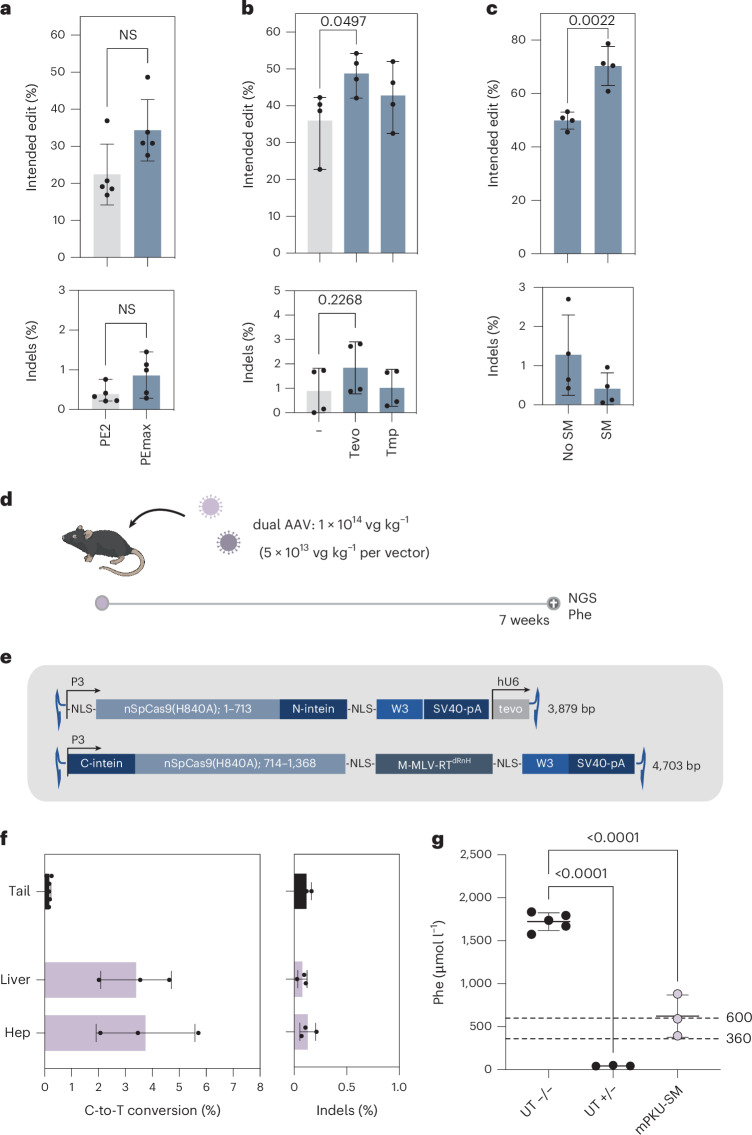
In vivo correction of *Pah*^*enu2*^ mice using AAV-mediated delivery of intein-split PEmax. **a**–**c**, Correction (top) and indel rates (bottom) in HEK293T cells with the integrated *Pah*^*enu2*^ locus, transfected with PE2 versus PEmax (*n* = 5) (**a**), with unmodified (grey) versus tevopreQ_1_ (tevo) modified versus tmpknot (tmp) modified pegRNAs (Supplementary Table 1) (*n* = 4) (**b**) or with tevopreQ1-mPKU-2.1 versus tevopreQ1-mPKU-SM (*n* = 4) (**c**). Values represent mean ± s.d. of independent biological replicates. Means were compared using unpaired two-sided Student’s *t*-tests. NS, not significant, *P* > 0.05. **d**, Schematic illustration of the experimental outline of the analysis in adult PKU mice. **e**, Schematic illustration of the AAV constructs selected for in vivo experiments. Indicated are AAV genome lengths including inverted terminal repeats (ITRs) in base pairs (bp). W3, truncated version of the posttranscriptional regulatory element of woodchuck hepatitis virus; nSpCas9, nickase of *Streptococcus pyogenes* Cas9; tevo, trimmed engineered pegRNA; M-MLV-RT^dRnH^, M-MLV RT-delta RNase H; SV40-pA, simian virus 40 poly-adenylation signal; NLS, nuclear localization signal. **f**, Correction rates in PKU mice (*n* = 3 mice). Editing rates were assessed in lysed tail tissue, liver tissue and isolated hepatocytes. Values depict mean ± s.d. Hep, hepatocytes. **g**, Phe levels at the experimental end point of untreated homozygous *Pah*^*enu2*^ mice (UT −/−) (*n* = 5), untreated heterozygous mice (UT +/−) (*n* = 3) and homozygous treated mice (*n* = 3). Dotted lines indicate recommended therapeutic thresholds for Phe levels in adults (600 μmol l^−1^) or in children/during pregnancy (360 μmol l^−1^)^29,57^. Values represent mean ± s.d. of independent biological replicates and were analysed using an ordinary one-way analysis of variance using Dunnett’s multiple comparisons test.

We exchanged PE2 with PEmax^23^—a PE variant with optimized codon usage and nuclear localization signal-linker designs—and incorporated pseudo-knot structures to the 3′ end of the pegRNA for protection from exonucleases (epegRNAs)^24,25^. In addition, we modified the RTT sequence of the pegRNA to co-introduce a silent mutation in the GG sequence of the protospacer adjacent motif (PAM), preventing retargeting of the locus once the edit is installed (Supplementary Fig. 1b). When transfected into HEK293T cells that contain an ectopic copy of the murine *Pah*^*enu2*^ locus (Supplementary Fig. 1a), the adapted editing components increased correction rates and reduced insertion–deletion (indel) rates to background (Fig. 1a–c).

Next, we assembled AAV vectors for in vivo delivery of the optimized PE components (PEmax + tevopreQ_1_-mPKU-SM) into *Pah*^*enu2*^ mice. As the size of the PE exceeds the packaging capacity of AAV, we used the intein-split system to split Cas9 and express the PE from two separate AAVs^14,15,17^. Testing two intein-split designs for PEmax (1153/1154 and 713/714), we observed comparable activity in HEK293T cells (Supplementary Fig. 1c). However, we selected the 713/714 variant for our in vivo experiments as it facilitates the generation of AAV constructs that are more equal in size when the non-essential RNaseH domain of the reverse transcriptase is removed and the pegRNA is positioned on the vector containing the N-intein. The tevopreQ_1_-mPKU-SM pegRNA was cloned downstream of the human U6 promoter, and N- and C-terminal fragments of PEmax were cloned between the liver-specific P3 promoter^26^ and a 3′ UTR containing the W3 post-transcriptional regulatory element^27^ and the simian virus (SV40)-polyA tail (Fig. 1d and Supplementary Fig. 1d). Recombinant AAV2 genomes were then packaged into hepatotropic AAV serotype 9 capsids (AAV2/9) and systemically administered to *Pah*^*enu2*^ mice via the tail vein in a 1:1 ratio at a final dose of 1 × 10^14^ vg kg^−1^ (Fig. 1e). Analysis of isolated hepatocytes by next-generation sequencing (NGS) after a period of 7 weeks revealed 4.6% correction of the *Pah*^*enu2*^ mutation (Fig. 1f), which resulted in a reduction of blood Phe levels from over 1,500 µmol l^−1^ to 623.7 µmol l^−1^ (Fig. 1g and Supplementary Fig. 2a). In line with previous genome editing studies that used AAV9 vectors in combination with the hepatocyte-specific P3 promoter, we found that editing was largely limited to hepatocytes^8,26,28^ (Supplementary Fig. 2b). Furthermore, we did not detect unintended editing in the liver of treated mice at the top five experimentally identified off-target binding sites of the mPKU-SM pegRNA^17^ (Supplementary Fig. 2c,d).

### Prime editing in the liver using LNP-mediated RNA delivery

While *Pah*^*enu2*^ correction rates with optimized AAV vectors and prime editing components were substantially increased compared to our initial study^22^, they were not sufficient to reduce Phe levels below the therapeutic threshold (600 µmol l^−1^ for adult PKU patients and 360 µmol l^−1^ for children and pregnant women)^29^. In addition, AAV doses of 1 × 10^14^ vg kg^−1^ can lead to severe immune reactions^30,31^, and prolonged PE expression from AAV vectors could lead to an accumulation of off-target mutations or induction of immune responses to the bacterial Cas9 or viral reverse transcriptase.

**Fig. 2:**
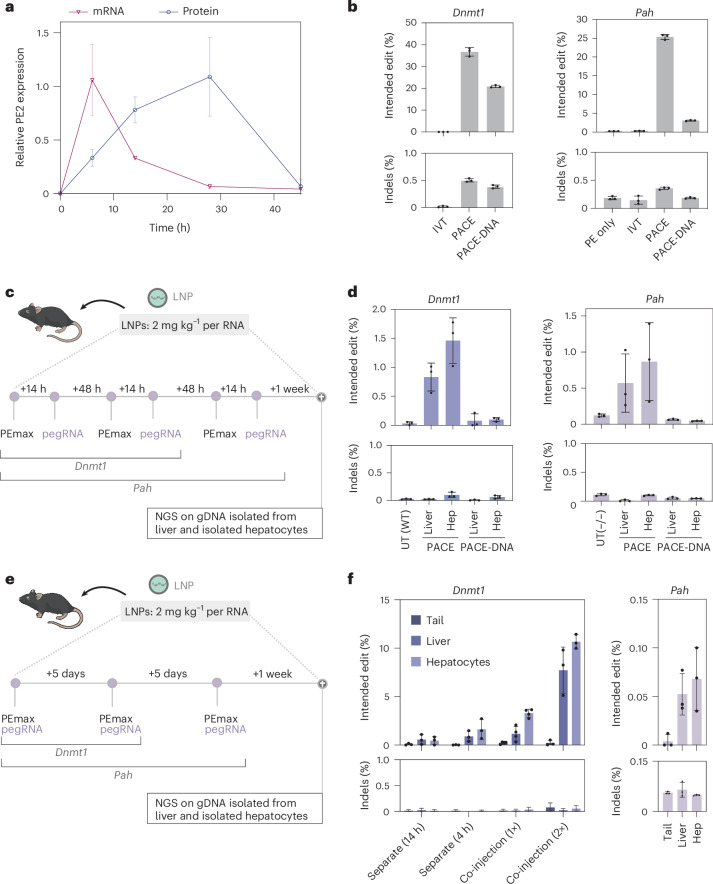
In vivo prime editing using RNA–LNP delivery. **a**, Expression kinetics of PE mRNA and protein (*n* = 2 mice). Relative values were normalized to the respective housekeeping gene and the average observed peak expression (6 and 28 h.p.i., respectively). **b**, In vitro editing rates at the *Dnmt1* locus (left) and *Pah*^*enu2*^ locus (right) after electroporation of HEK293T reporter cells with modified pegRNAs. IVT, in vitro transcribed. Values represent mean ± s.d. of three independent biological replicates. **c**,**e**, Schematic illustration of the experimental set-ups. Mice were dosed with PEmax mRNA 14 h before pegRNA injection for a total of two injection rounds targeting the *Dnmt1* locus and three injections rounds for the *Pah* locus (**c**), or PEmax was co-delivered with the respective pegRNA twice for the *Dnmt1* locus and three times for the *Pah* locus (**e**). **d**, In vivo editing rates of the *Dnmt1* locus in C57BL/6J mice (left; *n* = 3) and of the *Pah*^*enu2*^ locus in homozygous (−/−) B6 *Pah*^*enu2*^ mice (right; *n* = 3 with unmodified pegRNA and phosphonoacetate (PACE)-DNA-modified pegRNA, *n* = 6 with PACE-modified pegRNA). **f**, In vivo editing rates at the *Dnmt1* locus in C57BL/6J mice (left; *n* = 4 for 1× co-injection and *n* = 3 for the other combinations) and at the *Pah*^*enu2*^ locus in homozygous (−/−) B6 *Pah*^*enu2*^ mice (right; *n* = 3). In **a**, **b**, **d** and **f**, values represent mean ± s.d. of independent biological replicates.

In previous studies, LNP-mediated mRNA and sgRNA delivery has been used for transient genome editing with Cas9 nucleases and base editors^8,28,32–36^. To assess whether a similar approach is feasible for prime editing, we generated nucleoside-modified mRNA^37,38^ encoding PE2 and PEmax and packaged it into LNP. Confirming transient liver expression after systemic delivery of 2 mg kg^−1^ mRNA–LNP, we observed a peak in PE mRNA levels at 6 h post injection (h.p.i.) and a peak in protein levels at 24 h.p.i., whereas at 45 h.p.i. neither PE mRNA nor PE protein levels were detectable anymore (Fig. 2a and Supplementary Fig. 3a,b). Next, we chemically synthesized the tevopreQ_1_-mPKU-SM pegRNA targeting the *Pah*^*enu2*^ locus, and a pegRNA that installs a G-to-C edit at the *Dnmt1* locus. This pegRNA has previously been used for in vivo prime editing in the liver using AAV- and adenoviral-mediated delivery^17^. To protect pegRNAs from exonucleases, they were modified with 2′-O-methyl-3′-phosphorothioate at the 5′ end and 2′-O-methyl-3′-phosphonoacetate at the 3′ end^39^. In addition, we generated pegRNAs in which the RTT was substituted with deoxyribose nucleotides for protection from endonucleases (DNA-mod pegRNAs)^40^ (Supplementary Table 4). Confirming functionality of the synthesized pegRNAs, co-electroporation with PEmax mRNA into HEK reporter cells resulted in 25% editing at the *Pah*^*enu2*^ site and 37% editing at the *Dnmt1* site using non-DNA modified pegRNAs, and 3% editing at the *Pah*^*enu2*^ site and 21% editing at the *Dnmt1* site using DNA-modified pegRNAs (Fig. 2b).

We then administered PEmax mRNA and pegRNA into mice using a repeated dosing scheme, in which a dose of 2 mg kg^−1^ LNP containing PEmax mRNA was followed 14 h later by a dose 2 mg kg^−1^ LNP containing the respective pegRNA. Although this dosing scheme ensured the presence of PE protein at the time of pegRNA delivery (Fig. 2a), editing rates remained at 1.4% at the *Dnmt1* locus (after two dosing rounds) and at 0.4% at the *Pah*^*enu2*^ locus (after three dosing rounds; Fig. 2c,d). Hypothesizing that toxicity or innate immune stimulation from the initial mRNA–LNP dose might have prevented hepatocytes from taking up the pegRNA–LNP^19^, we tested additional dosing regimens delivering 2′-*O*-methyl-3′-phosphorothioate end-protected pegRNA–LNP targeting *Dnmt1* simultaneously, 4 h or 14 h after the mRNA–LNP dose. It is worth noting that NGS analysis showed highest editing in mice injected simultaneously with mRNA–LNP and pegRNA–LNP (3.5% editing after a single dose and 10.5% editing when mice are re-dosed after 5 days; Fig. 2e,f). Therefore, we next also treated PKU mice with this dosing regimen. However, even though mice were re-dosed three times in a 5 day interval, editing rates remained marginal (0.1%; Fig. 2e,f).

### In vivo correction of *Pah*^*enu2*^ mice using PEmax mRNA–LNP

**Fig. 3:**
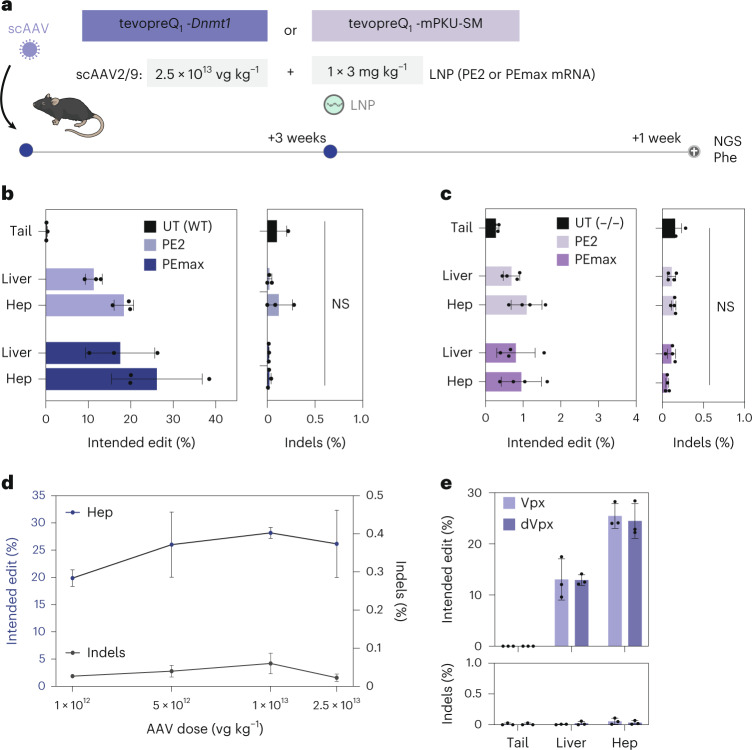
In vivo prime editing using pegRNA-AAV and PE mRNA–LNP. **a**, Schematic illustration of the experimental set-up. **b**, Editing rates (left) and indel rates (right) at the *Dnmt1* locus in untreated tail tissue or mice treated with a single dose of 3 mg kg^−1^ PE2 or PEmax mRNA–LNP (*n* = 3). **c**, *Pah*^*enu2*^ correction rates (left) and indel rates (right) of untreated tail tissue (*n* = 5) or mice treated with a single dose of 3 mg kg^−1^ PE2 or PEmax mRNA–LNP (*n* = 4). In **b** and **c**, values represent mean ± s.d. of independent biological replicates and were analysed for the indels using unpaired two-sided Student’s *t*-tests (*P* > 0.05) **d**, Editing rates (blue line, left *y* axis) and indel rates (grey bar, right *y* axis) of mice treated with one dose of 3 mg kg^−1^ PEmax mRNA–LNP and pretreatment of different doses of scAAV encoding the *Dnmt1-*targeting pegRNA (*n* = 3). Values represent mean ± s.e.m. **e**, Editing rates (top) and indel rates (bottom) of mice pretreated with scAAV encoding the *Dnmt1-*targeting pegRNA and with one dose of 2 mg kg^−1^ PEmax mRNA–LNP and active Vpx or inactive Vpx (dVpx) coding mRNA (*n* = 3). In **b**, **c** and **e**, values represent mean ± s.d. of independent biological replicates.

We hypothesized that prime editing efficiency in the RNA–LNP approach could have been limited by the low abundance and rapid degradation of the pegRNA. Therefore, we also tested an alternative strategy where only the PE was transiently delivered via mRNA–LNP and the pegRNA was expressed from a DNA construct delivered via AAV. PKU mice were first administered with 2.5 × 10^13^ vg kg^−1^ self-complementary AAV2/9 (scAAV2/9) encoding the tevopreQ_1_-mPKU-SM pegRNA (Supplementary Fig. 4a), followed by a treatment with PE2 mRNA–LNP or PEmax mRNA–LNP at a dose of 3 mg kg^−1^ (Fig. 3a). In addition, wild-type mice pretreated with an scAAV2/9 encoding the tevopreQ1-modified *Dnmt1* pegRNA were injected with a single dose of 3 mg kg^−1^ mRNA–LNP (Fig. 3a). While editing rates were relatively low at *Pah*^*enu2*^ locus (1.1% with PE2 and 1.0% with PEmax), at the *Dnmt1* locus we observed 17.7% editing with PE2 and 26.2% editing with PEmax (Fig. 3b,c). As expected for a treatment that installs a base change on genomic DNA, editing rates were not decreased when mice were analysed after 4 months compared to 1 week after PE delivery (Supplementary Fig. 4b). Moreover, editing efficiency did not decrease when the scAAV2/9 dose was reduced from 2.5 × 10^13^ vg kg^−1^ to 1 × 10^12^ vg kg^−1^ (26.2% versus 20%; Fig. 3d) or when mRNA–LNP levels were reduced from 3 mg kg^−1^ to 2 mg kg^−1^ (26.2% versus 24%; Fig. 3b,e). It is worth noting that recent studies suggested that accessibility of deoxyribonucleoside triphosphates could be a limiting factor for prime editing in slowly dividing cells such as resting T cells or haematopoietic stem and progenitor cells (HSPCs)^41,42^, prompting us to also test an approach where we co-delivered PEmax mRNA with an mRNA encoding VPX—a lentiviral factor that increases deoxyribonucleoside triphosphate levels in quiescent cells by targeting SAMHD1 for degradation. However, in our experimental set-up, we did not observe an increase in prime editing efficiency at the *Dnmt1* locus compared to control mice where mRNA encoding inactive VPX^Q76A^ was co-delivered with the PE (Fig. 3e).

**Fig. 4:**
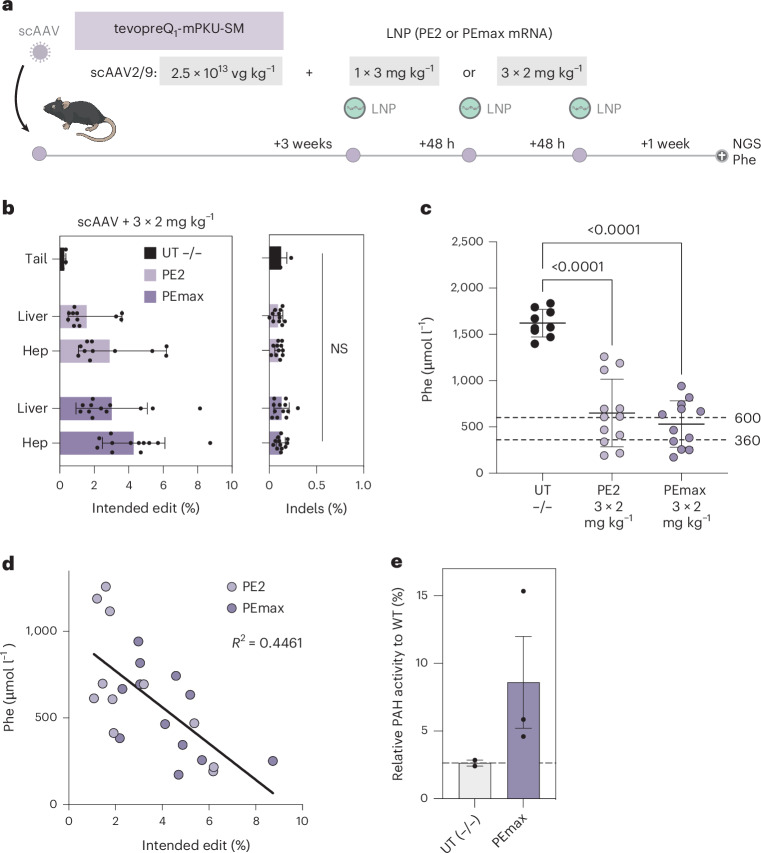
In vivo correction of *Pah*^*enu2*^ mice using pegRNA-AAV and PEmax mRNA–LNP. **a**, Schematic illustration of the experimental set-up. **b**, *Pah*^*enu2*^ correction rates (left) and indel rates (right) in untreated tail tissue (*n* = 5) or mice treated with scAAV2/9 expressing pegRNA tevopreQ1-mPKU-SM and PE2 or PEmax mRNA–LNP (*n* = 12). Values represent mean ± s.d. of independent biological replicates and for the indels were analysed using unpaired two-sided Student’s *t*-tests. **c**, Phe levels at the experimental end point of untreated homozygous B6 *Pah*^*enu2*^ mice (UT −/−; *n* = 8) and homozygous B6 *Pah*^*enu2*^ mice treated with scAAVs expressing the pegRNAs tevopreQ1-mPKU-SM and PE2 or PEmax mRNA–LNP (*n* = 12). Dotted lines indicate recommended therapeutic thresholds for Phe levels in adults (600 μmol l^−1^) or children/during pregnancy (360 μmol l^−1^)^29,57^. Values represent mean ± s.d. of independent biological replicates and were analysed using an ordinary one-way analysis of variance using a Dunnett’s multiple comparisons test. **d**, Correlation between editing rates in hepatocytes and Phe levels in PEmax (dark magenta) and PE2 (light magenta) treated mice at the experimental end points. *R*^2^, coefficient of determination. **e**, Enzyme activity of PAH in liver tissue lysates from untreated homozygous B6 *Pah*^*enu2*^ mice (*n* = 2) and homozygous B6 *Pah*^*enu2*^ mice treated with scAAV (pegRNA) and PEmax LNP–mRNA (*n* = 3). Values are depicted as relative values to PAH activity in wild-type mice. Values represent mean ± s.d. of independent biological replicates.

As correction rates at the *Pah*^*enu2*^ locus were not sufficient to substantially reduce Phe levels, we next re-dosed PKU mice that were pretreated with pegRNA-AAV three times with 2 mg kg^−1^ mRNA–LNP (Fig. 4a). It is worth noting that this redosing regimen increased editing rates up to 6.2% with PE2 (average 2.9%) and up to 8.7% with PEmax (average 4.3%; Fig. 4b), leading to a reduction of blood Phe levels below 600 µmol l^−1^ and a corresponding increase in PAH enzyme activity in the liver (Fig. 4c–e). Similar to the *Dnmt1* locus, also at the *Pah*^*enu2*^ locus editing rates did not substantially decrease when the scAAV2/9 dose was lowered from 2.5 × 10^13^ vg kg^−1^ to 5 × 10^12^ vg kg^−1^ (Supplementary Fig. 4c).

### In vivo correction of *Pah*^*enu2*^ mice using PE7 mRNA–LNP

**Fig. 5:**
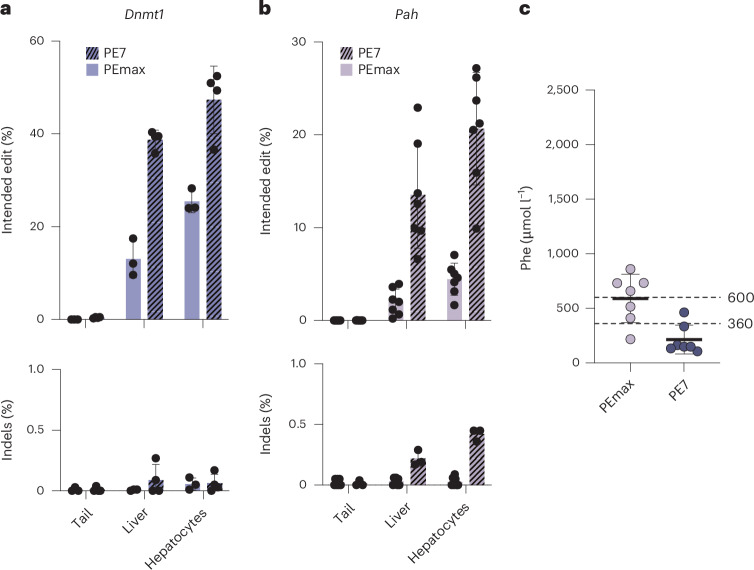
In vivo prime editing at *Dnmt1* locus and correction of *Pah*^*enu2*^ mice using pegRNA-AAV and PE7 mRNA–LNP. **a**, Editing rates (top) and indel rates (bottom) at the *Dnmt1* locus in mice pretreated with scAAV2/9 expressing the pegRNA tevopreQ1-*Dnmt1* at a dose of 2.5 × 10^13^ vg kg^−1^, which were subsequently injected once with 2 mg kg^−1^ LNP containing PEmax or PE7 mRNA (*n* = 4). **b**, *Pah*^*enu2*^ correction rates (top) and indel rates (bottom) of mice pretreated with scAAV2/9 expressing the pegRNA tevopreQ1-mPKU-SM4 at a dose of 2.5 × 10^13^ vg kg^−1^, which were subsequently injected three times with 2 mg kg^−1^ LNP containing PEmax or PE7 mRNA (*n* = 7). NGS was performed on isolated hepatocytes, whole liver and tail lysates. **c**, Phe levels at the experimental end point of homozygous B6 *Pah*^*enu2*^ mice (*n* = 7) from **b**. Dotted lines indicate recommended therapeutic thresholds for Phe levels in adults (600 μmol l^−1^) or in children/during pregnancy (360 μmol l^−1^)^29,57^. Values represent mean ± s.d. of independent biological replicates.

While in adults Phe levels below 600 µmol l^−1^ are considered therapeutic, in children and pregnant women Phe levels should be kept below 360 µmol l^−1^ to avoid neurotoxicity and cognitive impairment. Therefore, we attempted to further increase the correction efficiency of the *Pah*^*enu*^ allele by (1) designing a pegRNA that encodes for additional silent bystander mutations and that was computationally predicted by PRIDICT2.0 (ref. ^43^) to result in higher editing than pegRNA mPKU-SM (Supplementary Fig. 5a) and by (2) using a novel PE, PE7, in which the small RNA-binding factor La is fused to the PE to protect pegRNAs from exonucleases^44^. While the pegRNA encoding an additional silent mutation did not increase editing rates in vitro in cell lines (Supplementary Fig. 5b), or in vivo after three doses of 2 mg kg^−1^ mRNA–LNP with the full LNP-delivery approach (4.4% (Fig. 5b) versus 4.3% with pegRNA mPKU-SM (Fig. 4b)), the use of PE7 had a strong effect on editing efficiencies. We pretreated the mice with scAAV2/9 at a dose of 2.5 × 10^13^ vg kg^−1^ expressing the pegRNA tevopreQ1-Dnmt1 or the pegRNA tevopreQ1-mPKU-SM4 for targeting the *Dnmt1* locus or the *Pah*^*enu2*^, respectively. We obtained 47.4% editing at the *Dnmt1* locus after a single dose of 2 mg kg^−1^ mRNA–LNP (Fig. 5a) and 20.7% editing at the *Pah*^*enu2*^ locus after three doses of 2 mg kg^−1^ mRNA–LNP (Fig. 5b), which was sufficient to reduce Phe levels below the therapeutic threshold of 360 µmol l^−1^ (Fig. 5c).

**Fig. 6:**
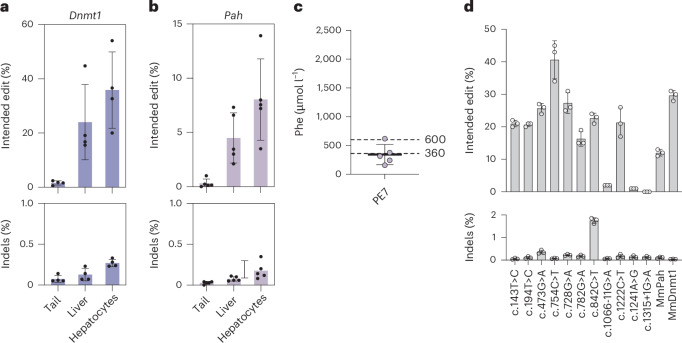
In vivo prime editing at *Dnmt1* locus and correction of *Pah*^*enu2*^ mice using full RNA–LNP delivery. **a**, G-to-C editing rates (top) and indel rates (bottom) at the *Dnmt1* locus in mice dosed twice with the full LNP–RNA approach using 2 mg kg^−1^ PE7 mRNA + 2 mg kg^−1^ pegRNA (*n* = 4). **b**, *Pah*^*enu2*^ correction rates (top) and indel rates (bottom) of mice dosed three times with the full LNP–RNA approach using 2 mg kg^−1^ PE7 mRNA + 2 mg kg^−1^ pegRNA (*n* = 5). In **a** and **b**, NGS was performed on isolated hepatocytes, whole liver and tail lysates. **c**, Phe levels at the experimental end point of homozygous B6 *Pah*^*enu2*^ mice dosed three times with 2 mg kg^−1^ PE7 mRNA + 2 mg kg^−1^ pegRNA (*n* = 5) from **b**. Dotted lines indicate recommended therapeutic thresholds for Phe levels in adults (600 μmol l^−1^) or in children/during pregnancy (360 μmol l^−1^)^29,57^. **d**, Prime editing rates in a K562 reporter cell line harbouring a DNA cassette containing the 11 most common human pathogenic PKU mutations as well as the *Pah*^*enu2*^ mutation and the *Dnmt1* locus for comparison. The top three predicted pegRNAs (PRIDICT2.0) were tested. Values represent mean ± s.d. of three independent biological replicates.

To next evaluate the PE7 system in a full RNA–LNP approach, we combined PE7 mRNA with synthetic La-accessible pegRNAs containing a polyU stretch (U*mU*mU*mUU) for La binding at their 3′ end. While this approach was less efficient than the dual AAV–LNP approach, treatment of mice with 2 mg kg^−1^ PE7 mRNA–LNP and 2 mg kg^−1^ *Dnmt1* pegRNA–LNP (2 doses with a 5 day interval between dosing) resulted in 35.9% editing (Fig. 6a), representing a 3.4-fold increase compared to the full RNA–LNP approach with PEmax (Fig. 2f). Moreover, in PKU mice treatment with 2 mg kg^−1^ PE7 mRNA–LNP and 2 mg kg^−1^ mPKU-SM pegRNA–LNP (three doses with a 5 day interval between dosing) resulted in 8.0% correction of the *Pah*^*enu2*^ mutation (Fig. 6b). This marks a substantial increase compared to the less than 0.5% editing observed with PEmax (Fig. 2d,f) and effectively reduced Phe levels to 342 µmol l^−1^.

PKU can be caused by various mutations in the *PAH* gene. We therefore next determined whether other pathogenic mutations can be corrected with similar editing efficiencies than the *Pah*^*enu2*^ allele. Using PRIDICT2.0, we predicted the most effective pegRNA designs for the 11 most common PKU mutations (Supplementary Fig. 5c) and selected the top three pegRNA designs per mutation for testing in K562 reporter cells, which are mismatch repair proficient and show pegRNA efficiency patterns similar to hepatocytes^43^. It is worth noting that for 8 out of the 11 tested mutations we observed editing rates that surpassed those achieved for the *Pah*^*enu2*^ mutation (Fig. 6d and Supplementary Fig. 5c), suggesting that in vivo prime editing using PE7 mRNA–LNP may offer a promising avenue for therapeutic application in PKU patients.

### Prime editing caused no off-target editing or liver damage

**Fig. 7:**
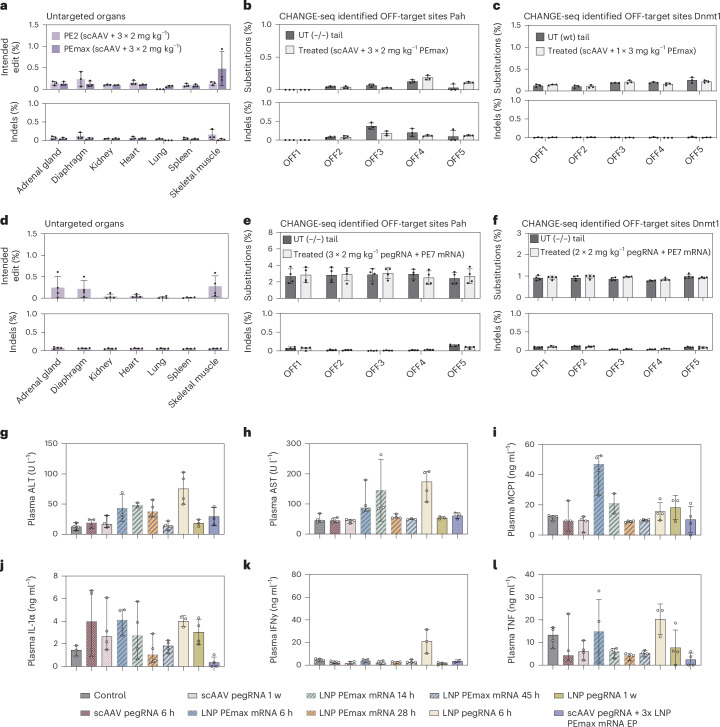
Assessment of off-target effects and liver toxicity in mice treated with pegRNA-AAV and mRNA–LNP. **a**,**d**, Editing rates at the *Pah*^*enu2*^ locus in different tissues. Mice were treated with scAAV encoding for the *Pah*^*enu2*^ targeting pegRNA and three doses of 2 mg kg^−1^ PE2 or PEmax (*n* = 3) (**a**) or treated with the full LNP–RNA approach using three doses of 2 mg kg^−1^ PE7 mRNA plus 2 mg kg^−1^ PKU pegRNA (*n* = 4) (**d**). **b**,**e**, Editing rates at CHANGE-seq identified off-target sites with the *Pah*^*enu2*^ targeting pegRNA^17^. **b**, Mice (*n* = 3) were treated with scAAV and 3 × 2 mg kg^−1^ PEmax mRNA–LNP. **e**, Mice (*n* = 4) were treated three times with 2 mg kg^−1^ PE7 plus 2 mg kg^−1^ PKU pegRNA. **c**,**f**, Editing rates at CHANGE-seq identified off-target sites^17^ of the *Dnmt1*-targeting pegRNA. Mice (*n* = 3) were treated with AAV and 1 × 3 mg kg^−1^ PEmax mRNA–LNP (**c**) or twice with 2 mg kg^−1^ pegRNA and 2 mg kg^−1^ PE7 mRNA–LNP (**f**). In **a**–**f**, values represent mean ± s.d. of independent biological replicates. In **b**, **c**, **e** and **f**, means were compared using Šídák’s multiple comparisons test, and no significant difference was found between treated and untreated samples (*P* > 0.05). **g**–**l**, Measured concentrations of markers for liver damage or inflammation in the plasma of treated mice at indicated time points: ALT (**g**), AST (**h**), monocyte chemoattractant protein-1 (MCP1) (**i**), Interleukin-1α (IL-1α) (**j**), Interferon-γ (IFNγ) (**k**) and tumour necrosis factor (TNF) (**l**); values represent mean ± s.d. independent biological replicates (control: *n* = 4 (**g**–**i**,**k**–**l**), *n* = 3 (**j**); scAAV-6h and scAAV-1w: *n* = 4 (**g**–**l**); LNP-6h: *n* = 4 (**h**–**l**), *n* = 3 (**g**); LNP-14h: *n* = 3 (**h**,**g**,**i**) and *n* = 4 (**j**–**l**); LNP-28h and LNP-45h: *n* = 4 (**g**–**l**); LNP_peg-6h: *n* = 3 (**i**–**l**), *n* = 4 (**g**–**h**); LNP_peg-1w: *n* = 3 (**g**–**h**), *n* = 4 (**i**–**l**); scAAV + LNP: *n* = 3 (**g**–**h**), *n* = 4 (**i**–**l**).

To assess whether prime editing using the dual AAV–LNP or full RNA–LNP approach was limited to the liver, DNA of treated mice was isolated from different organs and analysed by NGS. Consistent with previous mRNA–LNP biodistribution studies^45^, we did not observe substantial editing in any of the analysed tissues in *Pah*^*enu2*^-targeted or *Dnmt1*-targeted mice with the dual AAV–LNP or the full RNA–LNP approach (Fig. 7a,d and Supplementary Fig. 6a,b). Next, we assessed whether editing occurred at other sites in the genome in the liver of treated mice. However, when we performed targeted amplicon sequencing at the top five off-target binding sites of the two pegRNAs (identified by circularization for high-throughput analysis of nuclease genome-wide effects by sequencing (CHANGE-seq)^17^; Supplementary Figs. 2c and 6c), we did not observe editing above background—neither in the AAV–LNP approach nor in the full RNA–LNP approach (Fig. 7b,c,e,f).

Finally, we examined whether delivery of mRNA–LNP, pegRNA–LNP or pegRNA-AAV triggered liver toxicity or innate immune responses. A mild elevation of alanine transaminase (ALT) was observed 6 h after administration of 2 mg kg^−1^ PE mRNA–LNP or pegRNA–LNP, but levels returned to baseline levels at 45 h.p.i. or 1 week after administration, respectively (Fig. 7g,h). Similarly, transient increases in proinflammatory cytokines, such as MCP1 and IFNγ, were detected but normalized at later time points (Fig. 7i–l). When administering scAAV2/9 encoding the pegRNA at a dose of 2.5 × 10^13^ vg kg^−1^, we did not observe significant increases in ALT, aspartate aminotransferase (AST), or proinflammatory cytokines at any of the tested time points (Fig. 7g–l). In line with these observations, histological examination of the liver of treated mice did not reveal any obvious signs of tissue necrosis (Supplementary Fig. 6d).

## Discussion

While earlier studies have shown that PKU mice can be corrected by in vivo prime editing using viral-vector-mediated PE delivery^11,17^, the prolonged expression of the editing machinery poses limitations for clinical application of these approaches. Base editing, which is typically more efficient than prime editing, presents an interesting alternative and has recently been used to correct PKU mice using a transient RNA–LNP delivery approach^12,36,46^. However, most pathogenic PKU mutations are not targetable with current base editors, as they are not transition point mutations or contain additional target bases in the editing window that lead to undesirable bystander mutations. Another possible alternative to prime editing is classical gene addition therapy using AAV vectors to deliver a functional *Pah* gene into hepatocytes. While this approach achieved substantial reduction of Phe levels in PKU mice^47^, the episomal nature of AAV vectors and division of hepatocytes during tissue homeostasis and regeneration^13,48–50^ suggests that this method may not offer a permanent cure.

In this study, we developed transient in vivo prime editing approaches to correct PKU in mice. We initially started by delivering synthetic pegRNAs together with PEmax mRNA via LNP into the liver. At the *Dnmt1* locus, this approach led to 3.5% and 10.5% editing in hepatocytes after a single dose or two doses of RNA–LNP, respectively. These results align with a recent report showing 4.5% prime editing efficiency at the *Pcsk9* locus after a single dose and 8% after three doses of RNA–LNP^19^. However, at the *Pah*^*enu2*^ locus editing efficiencies were significantly lower (<0.5%) and insufficient to reduce Phe to therapeutic levels. Consequently, we next tested a dual AAV–LNP approach where the pegRNA was permanently expressed from an AAV vector and only the PE was transiently delivered via mRNA–LNP. This approach resulted in substantially increased editing rates, with 26.2% editing at the *Dnmt1* locus after a single dose of mRNA–LNP and 4.3% editing at the *Pah*^*enu2*^ locus after three doses of mRNA–LNP. While the editing rates at the *Pah*^*enu2*^ locus were sufficient to reduce Phe levels below the therapeutic threshold for adults (600 µmol l^−1^), they were insufficient to reach the therapeutic threshold for children (360 µmol l^−1^). This prompted us to test whether the novel PE7 PE variant, in which the small RNA-binding exonuclease protection factor La is fused to the reverse transcriptase, could further increase editing rates. Using PE7 mRNA–LNP in the dual AAV–LNP approach indeed resulted in 47.4% editing at the *Dnmt1* locus after a single dose and 20.7% correction of the *Pah*^*enu2*^ mutation after three doses, which was sufficient to reduce Phe levels below the therapeutic threshold of 360 µmol l^−1^. In our full LNP approach with PEmax, we had used pegRNAs that were already protected from exonucleases by chemical modifications on the 5′ and 3′ ends. However, these modifications are not necessarily fully protective, and Yan et al.^44^ hypothesized that in addition to the protection from exonucleases La could also enhance nuclear retention of the pegRNA and facilitate loading of the pegRNA on Cas9. Therefore, we next tested whether PE7 also enhances prime editing efficiency when PE7 mRNA was co-delivered with La-accessible synthetic pegRNAs. It is worth noting that this strategy resulted in 35.9% editing after two doses of RNA–LNP at the *Dnmt1* locus and 8.0% editing and therapeutic reduction of Phe levels below 360 µmol l^−1^ after three doses of RNA–LNP at the *Pah*^*enu2*^ locus.

In conclusion, our study presents two distinct strategies for transient prime editing in the liver. In the first approach the pegRNA is permanently expressed from AAV and only the PE is transiently delivered via mRNA–LNP. In the second approach both the pegRNA and the PE mRNA are transiently delivered via LNP. Although the dual AAV–LNP approach would require patients to be exposed to two different delivery vectors, it achieved substantially higher editing efficiencies when equivalent doses of mRNA–LNP were used. In the *Pah*^*enu2*^ PKU mouse model the use of PE7 in the full LNP approach resulted in correction efficiencies that were sufficient to reduce Phe levels below the therapeutic threshold, and comparison of prime editing efficiencies in K562 reporter cell lines suggests that this approach could also be effective for other human pathogenic PKU mutations. Given these results, the dual AAV–LNP approach may not be a good choice for PKU. However, for genetic diseases requiring higher editing efficiencies, the dual AAV–LNP approach may present a viable alternative. Future research will be critical in determining whether the dual-vector strategy is necessary for such cases or whether the full LNP approach can be further optimized to enhance editing efficiency, expanding its therapeutic potential for a broader spectrum of genetic disorders.

## Methods

### Generation of plasmids

To generate epegRNA plasmids, annealed spacer, scaffold and 3′ extension oligos (Supplementary Table 2) were cloned into pU6-tevopreq1-GG-acceptor (Addgene number 174038) by Golden Gate Assembly. For the generation of split-intein PEmax constructs, inserts were PCR amplified from the epegRNA plasmids and from pCMV-PEmax (Addgene number 174820) and inserted into the respective backbones (Addgene numbers 187181 and 117182) using HiFi DNA Assembly Master Mix (New England Biolabs, NEB). To generate PiggyBac PKU reporter plasmids, inserts with homology overhangs for cloning were ordered from IDT and cloned into the pPB-Zeocin backbone using HiFi DNA Assembly Master Mix (NEB). All PCRs were performed using Q5 High-Fidelity DNA Polymerase (NEB).

### Cell culture transfection and genomic DNA preparation

HEK293T (American Type Culture Collection, CRL-3216) or K562 (American Type Culture Collection, CCL-243) cells were maintained in Dulbecco’s modified Eagle’s medium (DMEM) plus GlutaMAX (Thermo Fisher Scientific) or Roswell Park Memorial Institute 1640 medium (Thermo Fisher Scientific), respectively, and supplemented with 10% (*v*/*v*) fetal bovine serum and 1% penicillin/streptomycin (Thermo Fisher Scientific) at 37 °C and 5% CO_2_. Cells were maintained at confluency below 90% (HEK293T) or below a density of 1.8 × 10^6^ cells per ml (K562) and seeded into 48-well cell culture plates (Greiner). About 1 × 10^5^ cells were transfected using 1.5 μl of Lipofectamine 2000 (Thermo Fisher Scientific) with 375 ng of PEmax and 125 ng of epegRNA or 250 ng of each AAV construct according to the manufacturer’s instructions. Unless otherwise noted, cells were incubated for 3 days, and genomic DNA was isolated by direct lysis. For nucleofections 0.5 pmol of the PEmax mRNA were used in a 1:1 molar ratio with the pegRNAs. Nucleofections of mRNA were carried out with one pulse of 1,400 mV and 20 ms pulse width using the Neon Nucleofector electroporation system by Invitrogen. After nucleofection, cells were cultured in 200 μl of DMEM plus GlutaMAX for 48 h before isolation of genomic DNA by direct lysis.

The unmodified pegRNA was generated by in vitro transcription as previously described, using a synthetic DNA fragment (Supplementary Table 4). The mRNA transcription template was prepared by PCR using a forward primer to introduce the correct T7 promoter sequence and a reverse primer to incorporate the poly(A) tail, with the synthetic DNA fragment serving as the PCR template. The mRNA was then transcribed from this PCR-derived template using the HiScribe T7 High Yield RNA Synthesis Kit (NEB).

### Generation of reporter cells by PiggyBac transposon

For generation of the PKU reporter cell lines with the PiggyBac transposon, 75,000 HEK293T or K562 cells were seeded into a 24-well culture plate (Greiner) and transfected the next day using Lipofectamine 2000 (Thermo Fisher Scientific) according to the manufacturer’s instructions. In brief, 1.5 μl of Lipofectamine was mixed with 23.5 μl of Opti-MEM medium, incubated at room temperature for 10 min and added to 225 ng of transposon plasmid and 25 ng of transposon helper plasmid (filled up to 25 μl of Opti-MEM). After 30 min of incubation at room temperature, cells were transfected. Three days after transfection, cells were enriched for 10 days using Zeocin (InvivoGen, 150 μg ml^−1^) selection with a final concentration of 0.1 μg ml^−1^.

### AAV production

Pseudo-typed AAV9 vectors (AAV2/9) were produced by co-transfection of packaging, capsid and helper plasmids (Addgene numbers 112865 and 112867), which were incubated for 5 days until collection. The cells were pelleted by low-speed centrifugation (1,500*g*, 15 min). The medium was then decanted, and the resulting cell pellet was resuspended in resuspension buffer (150 mM NaCl, 50 mM Tris–HCl), transferred into a Lysis Kit tube (Precellys Evolution) and mechanically disrupted according to the manufacturer’s instructions. Subsequently, the samples were lysed with benzonase (Sigma-Aldrich) at 37 °C for 30 min, followed by centrifugation at 5,000*g* for 1 h at 4 °C. The supernatant from this centrifugation step was combined with the supernatant from the previous step and precipitated using PEG8000 and NaCl solutions (1–2 days, 4 °C). The precipitated material was then pelleted by medium-speed centrifugation (5,000*g*, 1 h). The pellet was resuspended in resuspension buffer (final volume of 6 ml), and 1.5 ml of 5 M NaCl was added. The AAV particles were further purified by gradient centrifugation using OptiPrep (Sigma-Aldrich) according to the manufacturer’s instructions. Finally, the purified AAVs were concentrated using Vivaspin 20 centrifugal concentrators (VWR) following the manufacturer’s protocol.

Physical titres (vector genomes per millilitre) were determined using a Qubit 3.0 Fluorometer. In brief, the Qubit Fluorometer 3.0 (Life Technologies) was used to measure the concentrations (ng ml^−1^) of the extracted genomes by denaturation at 95 °C for 5 min, after which the readings were converted to vector genomes per millilitre using the genome’s molecular mass and Avogadro’s constant. The identity of the packaged genomes of each AAV vector was confirmed by Sanger DNA sequencing by Microsynth AG, testing 500 ng of denatured AAV using an AAV-genome-specific sequencing primer. AAV2/9 viruses were stored at −80 °C until use and diluted with phosphate-buffered saline (PBS; Thermo Fisher Scientific) if necessary.

### RNA synthesis and LNP encapsulation

mRNA production was performed as previously described^51^. In short, the coding sequences of the PEs were cloned into the mRNA production plasmid using HiFi DNA Assembly Master Mix (NEB). mRNAs were transcribed to contain 101 nucleotide-long poly(A) tails. m1Ψ-5′-triphosphate (TriLink) instead of UTP was used to generate modified nucleoside-containing mRNA. Capping of the in vitro transcribed mRNAs was performed co-transcriptionally using the trinucleotide cap1 analogue, CleanCap (TriLink). mRNA was purified by cellulose (Sigma-Aldrich) purification as previously described^52^. All mRNAs were analysed by agarose gel electrophoresis and were stored frozen at −20 °C. Synthetic pegRNAs were ordered and synthesized by Axolabs (peg-mod_1) or Agilent (peg-mod_2-4). LNP were formulated as previously described^53^. In short, an ethanolic solution of 1,2-distearoyl-*sn*-glycero-3-phosphocholine, cholesterol, a PEG lipid and an ionizable cationic lipid was rapidly mixed with an aqueous solution (pH 4) containing PE mRNA using an in-line mixer. The PEG and ionizable lipid used in this study are described in patent application WO 2017/004143. The resulting LNP formulation was dialysed overnight against 1× PBS, 0.2 μm sterile filtered and stored at −80 °C at a concentration of 1 μg μl^−1^ of total RNA. Encapsulation efficiencies of mRNA in the LNP were >97% as measured by the Quant-iT Ribogreen Assay (Life Technologies), and LNP sizes were below 80 nm as measured by a Malvern Zetasizer (Malvern Panalytical). Acuitas Therapeutics will provide the LNP formulation used in this study to any academic researcher who wishes to test and replicate our findings.

### Animal studies

Animal experiments were performed in accordance with protocols approved by the Kantonales Veterinäramt Zürich and in compliance with all relevant ethical regulations. B6.BTBR-*Pah*^*enu2*^ (strain number 029218) and C57BL/6J (strain number 000664) mice were housed in a pathogen-free animal facility at the Institute of Pharmacology and Toxicology of the University of Zurich. Mice were kept in a temperature- and humidity-controlled room on a 12 h light–dark cycle. Mice were fed a standard laboratory chow (Kliba Nafag number 3437 with 18.5% crude protein) and genotyped at weaning. Heterozygous *Pah*^*enu2*^ littermates were used as controls for physiological Phe concentrations in the blood (<120 μmol l^−1^). For sampling of blood for Phe determination, mice were fasted for 3 to 4 h, and the blood was collected from the tail vein. Adult mice were injected with 5–10 × 10^13^ vg kg^−1^ (AAV) or with 1–3 mg kg^−1^ RNA–LNP in a maximal volume of 150 µl via the tail vein. The selected AAV and RNA–LNP doses were based on the maximum injection volume for adults (150 μl of undiluted viral vectors via the tail vein). Mice were injected with AAVs and/or LNPs via the tail vein from 6 to 12 weeks of age.

### Primary hepatocyte isolation

Primary hepatocytes were isolated using a two-step perfusion method. Briefly, pre-perfusion with Hanks’ buffer (supplemented with 0.5 mM EDTA and 25 mM Hepes) was performed by inserting the cannula through the superior vena cava and cutting the portal vein. Next, livers were perfused at low flow for about 10 min with digestion buffer (low-glucose DMEM supplemented with 1 mM HEPES) containing freshly added Liberase (32 μg ml^−1^; Roche). Digestion was stopped using isolation buffer (low-glucose DMEM supplemented with 10% fetal bovine serum), and cells were separated from the matrix by gently pushing with a cell scraper. The cell suspension was filtered through a 100 μm filter (Corning), and hepatocytes were purified by two low-speed centrifugation steps (50*g* for 2 min).

### PCR amplification for deep sequencing

Genomic DNA from mouse tissues were isolated by direct lysis. Locus-specific primers were used to generate targeted amplicons for deep sequencing. First, input genomic DNA was amplified in a 10 μl reaction for 26 cycles using NEBNext High-Fidelity 2× PCR Master Mix (NEB). PCR products were purified using Sera-Mag magnetic beads (Cytiva) and subsequently amplified for six cycles using primers with sequencing adapters. Approximately equal amounts of PCR products from each sample were pooled, gel purified and quantified using a Qubit 3.0 fluorometer and the dsDNA HS Assay Kit (Thermo Fisher Scientific). Paired-end sequencing of purified libraries was performed on an Illumina MiSeq.

### NGS data analysis

Sequencing reads were demultiplexed using MiSeq Reporter (Illumina). The NGS data were collected and demultiplexed by Illumina NovaSeq Control software (v1.7) and MiSeq Control software (v3.1 and v4.0). Data analysis and visualization was performed using Python v3.9 and v3.10. Cutadapt (v3.1) was used to trim sequencing reads. For characterization of endogenous editing and unintended edits, CRISPResso2 (v2.2.7) was used^54^. Prime editing efficiencies were calculated as percentage of (number of reads containing only the desired edit)/(number of total reads). Indel yields were calculated as percentage of (number of indel-containing reads)/(total reads).

### Quantification of phenylalanine in the blood

Amino acids were extracted from a 3.2 mm dried blood sample using the Neomass AAAC Plus newborn screening kit (Labsystems Diagnostics). An ultra-high performance liquid chromatography system Nexera X2 coupled to an LCMS-8060 triple quadrupole mass spectrometer with electrospray ionization (Shimadzu) was used for the quantitative analysis of phenylalanine. LabSolutions v.6.115 and Neonatal Solution software (Shimadzu) were used for data acquisition and data analysis.

### Quantification of phenylalanine enzyme activity

Whole-liver extracts were analysed using isotope-dilution liquid chromatography–electrospray ionization–tandem mass spectrometry^55^. In brief, Phe (1 mmol l^−1^) was pre-incubated at 25 °C for 4 min, then Fe(NH_4_)_2_(SO_4_)_2_ (100 μmol l^−1^) was added, and incubation was continued for one more minute. After 5 min total pre-incubation time, BH_4_ (75 μmol l^−1^ for mouse tissue) was added to start the reaction. Between 2.5 and 20 μl (2–165 μg) of total protein lysate extracted from mouse liver tissue was used for activity measurements. The applied total protein amount depended on mouse liver tissue samples. It was generally higher for sample types with low activity where only little amounts of Tyr were produced. Initially, this was determined empirically by measuring a series of increasing total protein amounts for each sample type to determine the measurable linear range. Reaction time was 2 min; short incubation time reduces any possible chaperone-like effect of BH_4_. The amount of Tyr produced was determined by liquid chromatography with electrospray ionization–tandem mass spectrometry.

### Western blotting

Proteins were isolated from liver samples of treated and untreated mice. In brief, cells were lysed in RIPA buffer, supplemented with protease inhibitors (Sigma-Aldrich). Protein amounts were determined using the Pierce BCA Protein Assay Kit (Thermo Fisher). Equal amounts of protein (80 μg) were separated by SDS–PAGE (Thermo Fisher) and transferred to a 0.45 μm nitrocellulose membrane (Amersham). Membranes were incubated with mouse anti-Cas9 (1:1,000; catalogue number 14697T; Cell Signaling) and rabbit anti-GAPDH (1:10,000; catalogue number ab181602; abcam). Signals were detected by fluorescence using IRDye-conjugated secondary antibodies; IRDye 800CW Goat anti-Rabbit IgG Secondary Antibody (catalogue number 926-32211, LI-COR bio, 1:15,000) and IRDye 680RD Goat anti-Mouse IgG Secondary Antibody (catalogue number 926-68070, LI-COR bio, 1:15,000).

### RNA isolation and RT–qPCR

RNA was isolated from shock-frozen liver samples using the RNeasy Mini kit (Qiagen) according to the manufacturer’s instructions. RNA was reverse transcribed to cDNA using random primers and GoScript Reverse Transcriptase kit (Promega). Reverse transcription quantitative PCR (RT–qPCR) was performed using Firepol qPCR Master Mix (Solis BioDyne) and analysed by 7900HT Fast Real-Time PCR System (Applied Biosystems). Fold changes were calculated using the delta *C*_t_ method. Used primers are listed in Supplementary Table 3.

### Immunofluorescence

During liver perfusion, one liver lobe was tightened off using a silk suture thread (Fine Science Tools). Tissues were transferred to a 30% sucrose solution overnight at 4 °C and embedded in optimal cutting temperature (OCT) compound in cryomolds (Tissue-Tek) and frozen at −80 °C for at least 30 min. Frozen tissues were sectioned at 7 µm at −20 °C and mounted directly on Superfrost Plus slides (Thermo Fisher Scientific). Cryosections were counterstained with DAPI (Thermo Fisher Scientific) and mounted in VECTASHIELD mounting medium (Vector Labs). Two frozen sections per mouse per tissue were analysed. Mouse tissue was imaged using Zeiss Axioscope and Colibri 7 LED Illumination lighting system. Imaging conditions and intensity scales were matched for all images. Images were taken using Zeiss software Zen2 and analysed by Fiji ImageJ software (v1.51n)^56^.

### Histology

Livers were fixed using 4% paraformaldehyde (Sigma-Aldrich), followed by ethanol dehydration and paraffinization. Paraffin blocks were cut into 5-μm-thick sections, deparaffinized with xylene and rehydrated. Sections were haematoxylin–eosin-stained and examined for histopathological changes.

### Detection of plasma pro-inflammatory and damage markers

Blood was collected from the inferior vena cava using lithium–heparin-coated 0.5 ml tubes (MiniCollect) before liver perfusion. Samples were centrifuged at 2,000*g* for 10 min, and the supernatant was collected and stored at −20 °C until measurement. AST and ALT levels from all mouse samples were measured as routine parameters at the Division of Clinical Chemistry and Biochemistry at the University Children’s Hospital Zurich using Alinity ci-series. Pro-inflammatory cytokines were detected using LEGENDplex Mouse Inflammation panel (13-plex; Biolegend, catalogue number 740446, lot number B354399), a bead-based multiplex assay, according to the manufacturer’s instructions.

### Statistical analysis

Statistical analyses were performed using SciPy (1.6.3) amd GraphPad Prism 9.0.0 for macOS. Data are represented as biological replicates and are depicted as mean ± s.d. or s.e.m. as indicated in the corresponding figure legends. Likewise, sample sizes and the statistical tests used are described in detail in the respective figure legends. For all analyses, *P* < 0.05 was considered statistically significant.

### Reporting summary

Further information on research design is available in the Nature Portfolio Reporting Summary linked to this article.

## Supplementary information

Supplementary Information Supplementary Figs. 1–7, Notes 1 and 2 and Tables 1–4.

Reporting Summary

## Data Availability

All data associated with this study are present in the paper or the Supplementary information. Illumina sequencing data are available in the Sequence Read Archive under the accession number PRJNA947564 and PRJNA1258720.

## References

[1] Blau, N., Van Spronsen, F. J. & Levy, H. L. Phenylketonuria. *Lancet* **376**, 1417–1427 (2010). 20971365 10.1016/S0140-6736(10)60961-0

[2] Mitchell, J. J., Trakadis, Y. J. & Scriver, C. R. Phenylalanine hydroxylase deficiency. *Genet. Med.* **13**, 607–617 (2011). 21555948 10.1097/GIM.0b013e3182141b48

[3] Scriver, C. R. & Clow, C. L. Phenylketonuria: epitome of human biochemical genetics. *N. Engl. J. Med.* **303**, 1394–1400 (1980). 7432385 10.1056/NEJM198012113032404

[4] Hydery, T. & Coppenrath, V. A. A comprehensive review of pegvaliase, an enzyme substitution therapy for the treatment of phenylketonuria. *Drug Target Insights* **13**, 1177392819857089 (2019). 10.1177/1177392819857089 PMC6589953 31258325

[5] Martinez, M., Harding, C. O., Schwank, G. & Thöny, B. State-of-the-art 2023 on gene therapy for phenylketonuria. *J. Inherit. Metab. Dis.* **47**, 80–92 (2024). 37401651 10.1002/jimd.12651 PMC10764640

[6] Yin, H. et al. Therapeutic genome editing by combined viral and non-viral delivery of CRISPR system components in vivo. *Nat. Biotechnol.* **34**, 328–333 (2016). 26829318 10.1038/nbt.3471 PMC5423356

[7] Shedlovsky, A., McDonald, J. D., Symula, D. & Dove, W. F. Mouse models of human phenylketonuria. *Genetics* **134**, 1205–1210 (1993). 8375656 10.1093/genetics/134.4.1205 PMC1205587

[8] Villiger, L. et al. Treatment of a metabolic liver disease by in vivo genome base editing in adult mice. *Nat. Med.* **24**, 1519–1525 (2018). 30297904 10.1038/s41591-018-0209-1

[9] Richards, D. Y. et al. AAV-mediated CRISPR/Cas9 gene editing in murine phenylketonuria. *Mol. Ther. Methods Clin. Dev.* **17**, 234–245 (2019). 31970201 10.1016/j.omtm.2019.12.004 PMC6962637

[10] Zhou, L. et al. A universal strategy for AAV delivery of base editors to correct genetic point mutations in neonatal PKU mice. *Mol. Ther. Methods Clin. Dev.* **24**, 230–240 (2022). 35141352 10.1016/j.omtm.2022.01.001 PMC8803597

[11] Brooks, D. L. et al. Efficient in vivo prime editing corrects the most frequent phenylketonuria variant, associated with high unmet medical need. *Am. J. Hum. Genet* **110**, 2003–2014 (2023). 37924808 10.1016/j.ajhg.2023.10.005 PMC10716342

[12] Brooks, D. L. et al. Rapid and definitive treatment of phenylketonuria in variant-humanized mice with corrective editing. *Nat. Commun.* **14**, 3451 (2023). 37301931 10.1038/s41467-023-39246-2 PMC10257655

[13] Anzalone, A. V. et al. Search-and-replace genome editing without double-strand breaks or donor DNA. *Nature* **576**, 149–157 (2019). 31634902 10.1038/s41586-019-1711-4 PMC6907074

[14] Zheng, C. et al. A flexible split prime editor using truncated reverse transcriptase improves dual-AAV delivery in mouse liver. *Mol. Ther.* **30**, 1343–1351 (2022). 34998953 10.1016/j.ymthe.2022.01.005 PMC8899602

[15] Zhi, S. et al. Dual-AAV delivering split prime editor system for in vivo genome editing. *Mol. Ther.* **30**, 283–294 (2022). 34298129 10.1016/j.ymthe.2021.07.011 PMC8753371

[16] Newby, G. A. & Liu, D. R. In vivo somatic cell base editing and prime editing. *Mol. Ther.* **29**, 3107–3124 (2021). 34509669 10.1016/j.ymthe.2021.09.002 PMC8571176

[17] Böck, D. et al. In vivo prime editing of a metabolic liver disease in mice. *Sci. Transl. Med.* **14**, 9238 (2022). 10.1126/scitranslmed.abl9238 PMC7614134 35294257

[18] Liu, P. et al. Improved prime editors enable pathogenic allele correction and cancer modelling in adult mice. *Nat. Commun.* **12**, 2121 (2021). 33837189 10.1038/s41467-021-22295-w PMC8035190

[19] Chen, Z. et al. In vivo prime editing by lipid nanoparticle co-delivery of chemically modified pegRNA and prime editor mRNA. *GEN Biotechnol.* **2**, 490–502 (2023). 39850578 10.1089/genbio.2023.0045 PMC11756591

[20] Davis, J. R. et al. Efficient prime editing in mouse brain, liver and heart with dual AAVs. *Nat. Biotechnol.* **42**, 253–264 (2023). 37142705 10.1038/s41587-023-01758-z PMC10869272

[21] Qin, H. et al. Vision rescue via unconstrained in vivo prime editing in degenerating neural retinas. *J. Exp. Med.* **220**, e20220776 (2023). 36930174 10.1084/jem.20220776 PMC10037108

[22] Li, C. et al. In vivo HSC prime editing rescues sickle cell disease in a mouse model. *Blood* **141**, 2085–2099 (2023). 36800642 10.1182/blood.2022018252 PMC10163316

[23] Chen, P. J. et al. Enhanced prime editing systems by manipulating cellular determinants of editing outcomes. *Cell* **184**, 5635–5652.e29 (2021). 34653350 10.1016/j.cell.2021.09.018 PMC8584034

[24] Nelson, J. W. et al. Engineered pegRNAs improve prime editing efficiency. *Nat. Biotechnol.* **40**, 402–410 (2021). 34608327 10.1038/s41587-021-01039-7 PMC8930418

[25] Mathis, N. et al. Predicting prime editing efficiency and product purity by deep learning. *Nat. Biotechnol.* **41**, 1151–1159 (2023). 36646933 10.1038/s41587-022-01613-7 PMC7614945

[26] Viecelli, H. M. et al. Treatment of phenylketonuria using minicircle-based naked-DNA gene transfer to murine liver. *Hepatology* **60**, 1035–1043 (2014). 24585515 10.1002/hep.27104 PMC4449723

[27] Choi, J. H. et al. Optimization of AAV expression cassettes to improve packaging capacity and transgene expression in neurons. *Mol. Brain* **7**, 17 (2014). 24618276 10.1186/1756-6606-7-17 PMC3975461

[28] Rothgangl, T. et al. In vivo adenine base editing of PCSK9 in macaques reduces LDL cholesterol levels. *Nat. Biotechnol.* **39**, 949–957 (2021). 34012094 10.1038/s41587-021-00933-4 PMC8352781

[29] van Wegberg, A. M. J. et al. The complete European guidelines on phenylketonuria: diagnosis and treatment. *Orphanet J. Rare Dis.* **12**, 162 (2017). 29025426 10.1186/s13023-017-0685-2 PMC5639803

[30] Lek, A. et al. Death after high-dose rAAV9 gene therapy in a patient with Duchenne’s muscular dystrophy. *N. Engl. J. Med.* **389**, 1203–1210 (2023). 37754285 10.1056/NEJMoa2307798 PMC11288170

[31] Philippidis, A. Novartis confirms deaths of two patients treated with gene therapy Zolgensma. *Hum. Gene Ther.* **33**, 842–844 (2022). 36125439 10.1089/hum.2022.29216.bfs

[32] Musunuru, K. et al. In vivo CRISPR base editing of PCSK9 durably lowers cholesterol in primates. *Nature* **593**, 429–434 (2021). 34012082 10.1038/s41586-021-03534-y

[33] Finn, J. D. et al. A single administration of CRISPR/Cas9 lipid nanoparticles achieves robust and persistent in vivo genome editing. *Cell Rep.* **22**, 2227–2235 (2018). 29490262 10.1016/j.celrep.2018.02.014

[34] Yin, H. et al. structure-guided chemical modification of guide RNA enables potent non-viral in vivo genome editing. *Nat. Biotechnol.* **35**, 1179–1187 (2017). 29131148 10.1038/nbt.4005 PMC5901668

[35] Kenjo, E. et al. Low immunogenicity of LNP allows repeated administrations of CRISPR-Cas9 mRNA into skeletal muscle in mice. *Nat. Commun.* **12**, 1–13 (2021). 34880218 10.1038/s41467-021-26714-w PMC8654819

[36] Villiger, L. et al. In vivo cytidine base editing of hepatocytes without detectable off-target mutations in RNA and DNA. *Nat. Biomed. Eng.* **5**, 179–189 (2021). 33495639 10.1038/s41551-020-00671-z PMC7610981

[37] Pardi, N., Hogan, M. J., Porter, F. W. & Weissman, D. mRNA vaccines — a new era in vaccinology. *Nat. Rev. Drug Discov.* **17**, 261–279 (2018). 29326426 10.1038/nrd.2017.243 PMC5906799

[38] Andries, O. et al. N1-methylpseudouridine-incorporated mRNA outperforms pseudouridine-incorporated mRNA by providing enhanced protein expression and reduced immunogenicity in mammalian cell lines and mice. *J. Control. Release* **217**, 337–344 (2015). 26342664 10.1016/j.jconrel.2015.08.051

[39] Ryan, D. E. et al. Phosphonoacetate modifications enhance the stability and editing yields of guide RNAs for Cas9 editors. *Biochemistry* **62**, 3512–3520 (2023). 35436085 10.1021/acs.biochem.1c00768 PMC10734248

[40] Liu, B. et al. Targeted genome editing with a DNA-dependent DNA polymerase and exogenous DNA-containing templates. *Nat. Biotechnol.* **42**, 1039–1045 (2024). 37709915 10.1038/s41587-023-01947-w PMC12054351

[41] Levesque, S., Cosentino, A., Verma, A., Genovese, P. & Bauer, D. E. Enhancing prime editing in hematopoietic stem and progenitor cells by modulating nucleotide metabolism. *Nat. Biotechnol.* **43**, 534–538 (2025). 38806736 10.1038/s41587-024-02266-4 PMC13109851

[42] Liu, P. et al. Increasing intracellular dNTP levels improves prime editing efficiency. *Nat. Biotechnol.* **43**, 539–544 (2025). 39322763 10.1038/s41587-024-02405-x PMC12092096

[43] Mathis, N. et al. Machine learning prediction of prime editing efficiency across diverse chromatin contexts. *Nat. Biotechnol.*10.1038/s41587-024-02268-2 (2024). 38907037 10.1038/s41587-024-02268-2 PMC7617539

[44] Yan, J. et al. Improving prime editing with an endogenous small RNA-binding protein. *Nature* **628**, 639–647 (2024). 38570691 10.1038/s41586-024-07259-6 PMC11023932

[45] Witzigmann, D. et al. Lipid nanoparticle technology for therapeutic gene regulation in the liver. *Adv. Drug Deliv. Rev.* **159**, 344–363 (2020). 32622021 10.1016/j.addr.2020.06.026 PMC7329694

[46] Brooks, D. L. et al. A base editing strategy using mRNA-LNPs for in vivo correction of the most frequent phenylketonuria variant. *Hum. Genet. Genomics Adv.* **5**, 100253 (2024). 10.1016/j.xhgg.2023.100253 PMC10800763 37922902

[47] Kaiser, R. A. et al. Use of an adeno-associated virus serotype Anc80 to provide durable cure of phenylketonuria in a mouse model. *J. Inherit. Metab. Dis.* **44**, 1369–1381 (2021). 33896013 10.1002/jimd.12392 PMC9291745

[48] Duncan, A. W., Dorrell, C. & Grompe, M. Stem cells and liver regeneration. *Gastroenterology* **137**, 466 (2009). 19470389 10.1053/j.gastro.2009.05.044 PMC3136245

[49] Walton, R. T., Hsu, J. Y., Joung, J. K. & Kleinstiver, B. P. Scalable characterization of the PAM requirements of CRISPR–Cas enzymes using HT-PAMDA. *Nat. Protoc.* **16**, 1511–1547 (2021). 33547443 10.1038/s41596-020-00465-2 PMC8063866

[50] Grieger, J. C. & Samulski, R. J. Packaging capacity of adeno-associated virus serotypes: impact of larger genomes on infectivity and postentry steps. *J. Virol.* **79**, 9933 (2005). 16014954 10.1128/JVI.79.15.9933-9944.2005 PMC1181570

[51] van de Ven, K. et al. A universal influenza mRNA vaccine candidate boosts T cell responses and reduces zoonotic influenza virus disease in ferrets. *Sci. Adv.* **8**, eadc9937 (2022). 36516261 10.1126/sciadv.adc9937 PMC9750153

[52] Baiersdörfer, M. et al. A facile method for the removal of dsRNA contaminant from in vitro-transcribed mRNA. *Mol. Ther. Nucleic Acids* **15**, 26–35 (2019). 30933724 10.1016/j.omtn.2019.02.018 PMC6444222

[53] Conway, A. et al. Non-viral delivery of Zinc Finger Nuclease mRNA enables highly efficient in vivo genome editing of multiple therapeutic gene targets. *Mol. Ther.* **27**, 866–877 (2019). 30902585 10.1016/j.ymthe.2019.03.003 PMC6453547

[54] Clement, K. et al. CRISPResso2 provides accurate and rapid genome editing sequence analysis. *Nat. Biotechnol.* **37**, 224–226 (2019). 30809026 10.1038/s41587-019-0032-3 PMC6533916

[55] Heintz, C., Troxler, H., Martinez, A., Thöny, B. & Blau, N. Quantification of phenylalanine hydroxylase activity by isotope-dilution liquid chromatography-electrospray ionization tandem mass spectrometry. *Mol. Genet Metab.* **105**, 559–565 (2012). 22300847 10.1016/j.ymgme.2011.12.025

[56] Schindelin, J. et al. Fiji: an open-source platform for biological-image analysis. *Nat. Methods* **9**, 676–682 (2012). 22743772 10.1038/nmeth.2019 PMC3855844

[57] Vockley, J. et al. Phenylalanine hydroxylase deficiency: diagnosis and management guideline. *Genet. Med.* **16**, 188–200 (2014). 24385074 10.1038/gim.2013.157

