# Supplementary material for: Treatment of a metabolic liver disease in mice with a transient prime editing approach
Source: Nat Biomed Eng. 2025 May 20;9(10):1705–18. doi: 10.1038/s41551-025-01399-4 (PMC12532708; doi:10.1038/s41551-025-01399-4)
Supplement: Supplementary file 1 — Supplementary Figs. 1–7, Notes 1 and 2 and Tables 1–4. [file 41551_2025_1399_MOESM1_ESM.pdf]

nature biomedical engineering

Supplementary information https://doi.org/10.1038/s41551-025-01399-4

Treatment of a metabolic liver disease in mice with a transient prime editing approach

In the format provided by the authors and unedited

Supplementary Material

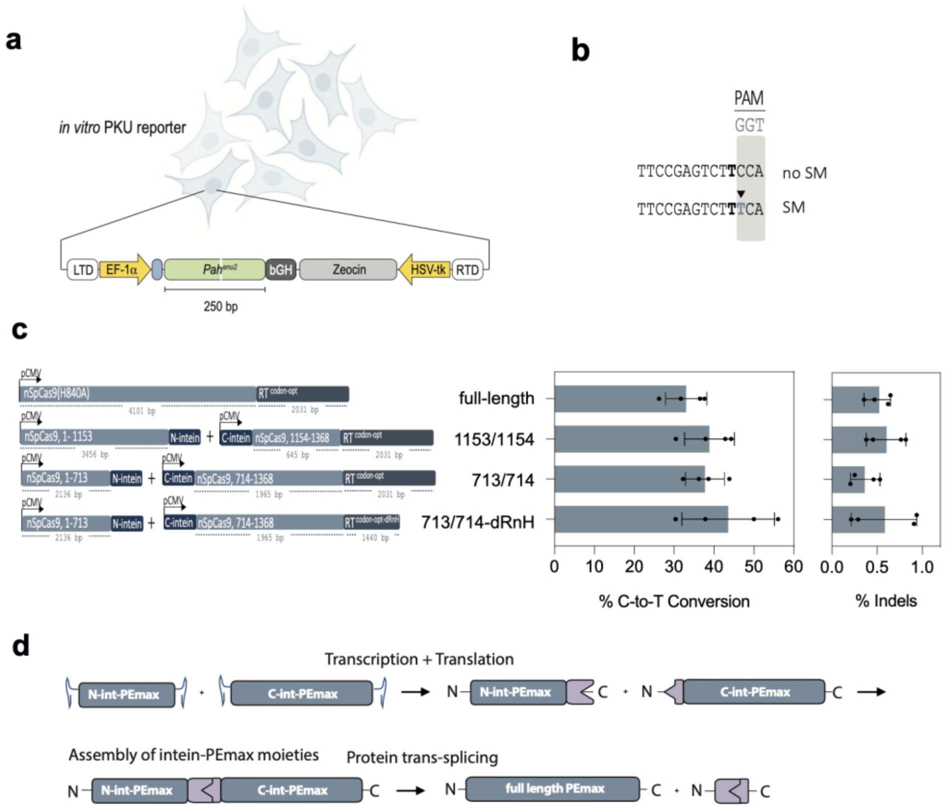

Figure S1 | *In vitro* optimization of PE components for correction of the disease-causing T-to-C mutation at the *Pah*^{enu2} locus. (**a**) Schematic representation of HEK293T or K562 *Pah*^{enu2} reporter cell lines, generated using the PiggyBac transposon system. The pegRNA binding site is indicated by a white line. RTD, right and LTD, left terminal domains; EF-1a, Human elongation factor-1 alpha promoter, *Pah*, Phenylalanine hydroxylase; bGH, bovine growth hormone polyadenylation signal; HSV-tk, herpes simplex virus thymidine kinase promoter; elements are not depicted to scale. (**b**) Schematic representation of the RTT in the pegRNAs, mPKU-2.1 (noSM) or mPKU-SM (SM). Bold letter indicates the base that corrects the pathogenic mutation. Black arrowhead indicates the site of the silent mismatch (SM) in the reverse transcriptase template (RTT). The part of the RTT complementary to the PAM is highlighted in grey. (**c**) Schematic representation of intein-split PEmax constructs (left panel) and editing rates with different variants tested in HEK293T cells with the integrated *Pah*^{enu2} locus. dRnH: PEmax without the RnaseH domain in the RT. Percentage of intended C-to-T conversions (middle panel) and indels (right panel). Values represent mean +/- s.d. of four independent biological replicates. (**d**) Schematic of the mechanism of the intein-split trans-splicing system.

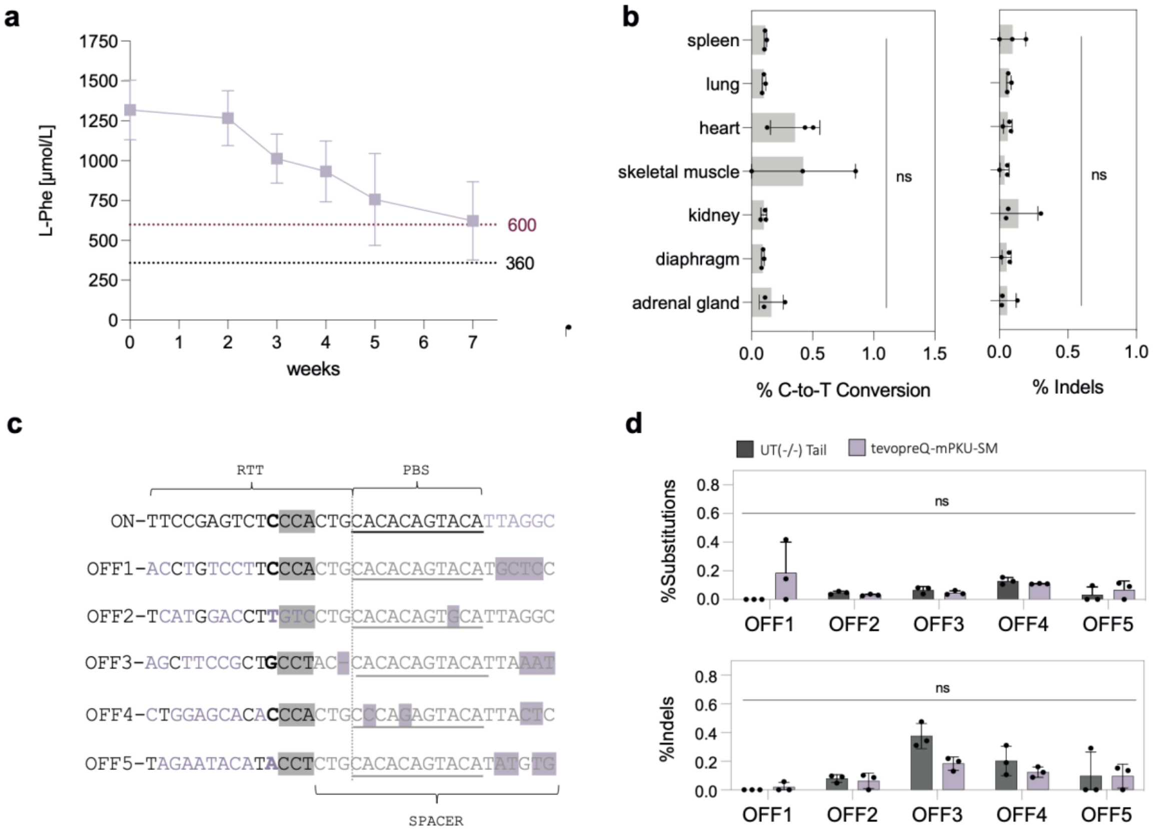

Figure S2 | *In vivo* prime editing using dual-AAV-mediated PE delivery in B6 Pah^{enu2} mice.

(**a**) Change of Phe levels over time in (n=3) animals treated with AAVs encoding for 713/714 PEmax-dRnH and tevopreQ$_1$-mPKU-SM. Values represent mean +/- s.d. of independent biological replicates. (**b**) C-to-T conversion rates (left panel) and indel rates (right panel) at the *Pah^{enu2}* locus in tissues other than the liver from animals treated with 1x10^{14} vg/kg AAV encoding for PEmax. Editing rates were assessed by targeted amplicon sequencing of whole tissue lysates. Means of n=3 independent biological replicates were compared using an ordinary one-way ANOVA using Šídák's multiple comparisons test. (**c-d**) Targeted amplicon sequencing of the top five off-target sites of the tevopreQ1-mPKU-SM pegRNA identified by CHANGE-seq in Böck et al.[19] (**c**) Indicated are the spacer, the primer binding site (underlined, PBS) and reverse transcriptase template (RTT) of the target site (ON) and 5 off-target sites (OFF1-OFF5) (spacer; gray letters). Mismatches to the target site are in purple (letters; mismatches to RTT, highlighted letters; mismatches to spacer). The bases complementary to the PAM site are highlighted in grey. The site of the DNA nick is indicated by a dotted line. Position of target-nucleotide indicated in bold letter. (**d**) Indel rates and substitution rates were quantified for tail tissue from untreated homozygous Pah^{enu2} animals (UT (-/-) Tail) and isolated hepatocytes from animals treated with dual-AAV with tevopreQ1-mPKU-SM. Means were compared using an ordinary one-way ANOVA using Šídák's multiple comparisons test. (b, d) Values depict mean +/- s.d. of n=3 independent biological replicates (ns, not significant, P > 0.05).

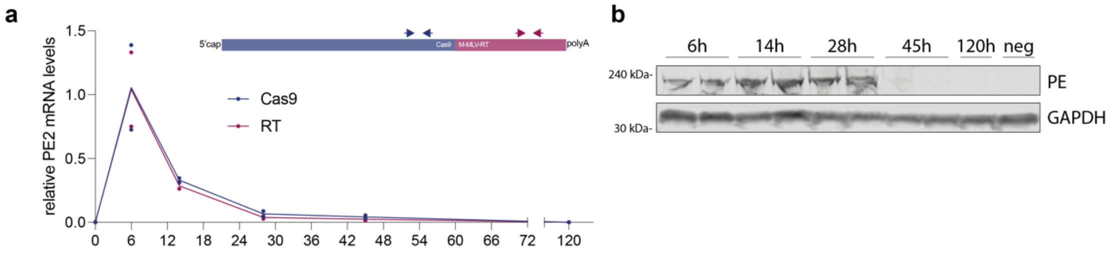

Figure S3 | Expression kinetics of the PE after mRNA-LNP delivery into the liver. (**a**) Expression kinetics of PE mRNA delivered via LNP at a dose of 2 mg/kg. Relative values were normalized to the expression of the housekeeping gene *Rplp0* and the average observed peak expression at 6 h.p.i. Each value represents mean of two individual biological replicates. The prime editor transcript was quantified separately using two targeting primers, where one binds the Cas9 (blue) and the other the RT (magenta) domain of the prime editor transcript. (**b**) Expression kinetics of the PE protein. Relative values were normalized to the expression of the housekeeping gene *Gapdh* and the average observed peak expression at 28 h.p.i. Each value represents mean of two individual biological replicates. The full western plot image is shown in supplementary note 2.

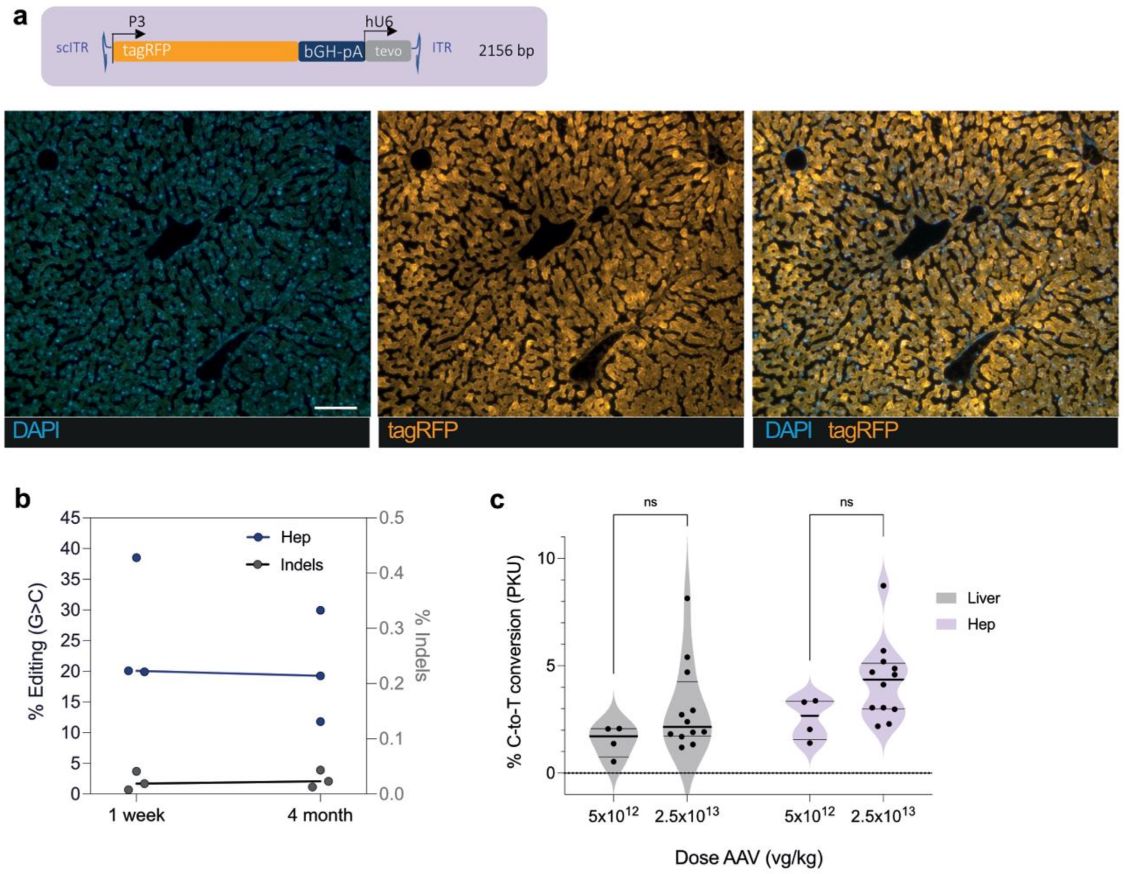

Figure S4 | *In vivo* prime editing rates in mice treated with pegRNA-AAV and PEmax mRNA-LNP. (**a**) Illustration of the self-complementary (sc) AAV genome expressing tevopreQ1-modified pegRNAs. scITR: self-complementary inverted terminal repeat; P3: liver-specific P3 promoter; tagRFP: tag red fluorescent protein; bGH-pA: bovine growth hormone polyadenylation signal, hU6: human U6 promoter, tevo: trimmed evopreQ_1-modified pegRNA (epegRNA). tagRFP expressing cells in the mouse liver 3 weeks after injection of scAAV at a dose of 2.5×10^{13} vg/kg. Scale bar: 50 µm. (**b**) Editing rates at the *Dnmt1* locus 1 week and 4 months after LNP-PEmax mRNA injection. Animals were pre-treated with the same scAAV-pegRNA dose as in (a). Lines connect medians and dots represent individual animals. Correction rates in hepatocytes (Hep) are shown in dark blue on the the left y-axis. Indel rates are shown in grey on the right y-axis. (**c**) Comparison of C-to-T conversion rates at the *Pah*^{enu2} locus using pretreatment with either 5×10^{12} (n=4) or 2.5×10^{13} (n=12) vg/kg scAAV expressing tevopreQ_1-mPKU-SM with subsequent three-times redosing of LNP expressing PEmax. Individual data points and medians are depicted from whole liver isolates and primary isolated hepatocytes of the same animals. Values were compared using Šídák's multiple comparisons test (ns, not significant, $P > 0.05$).

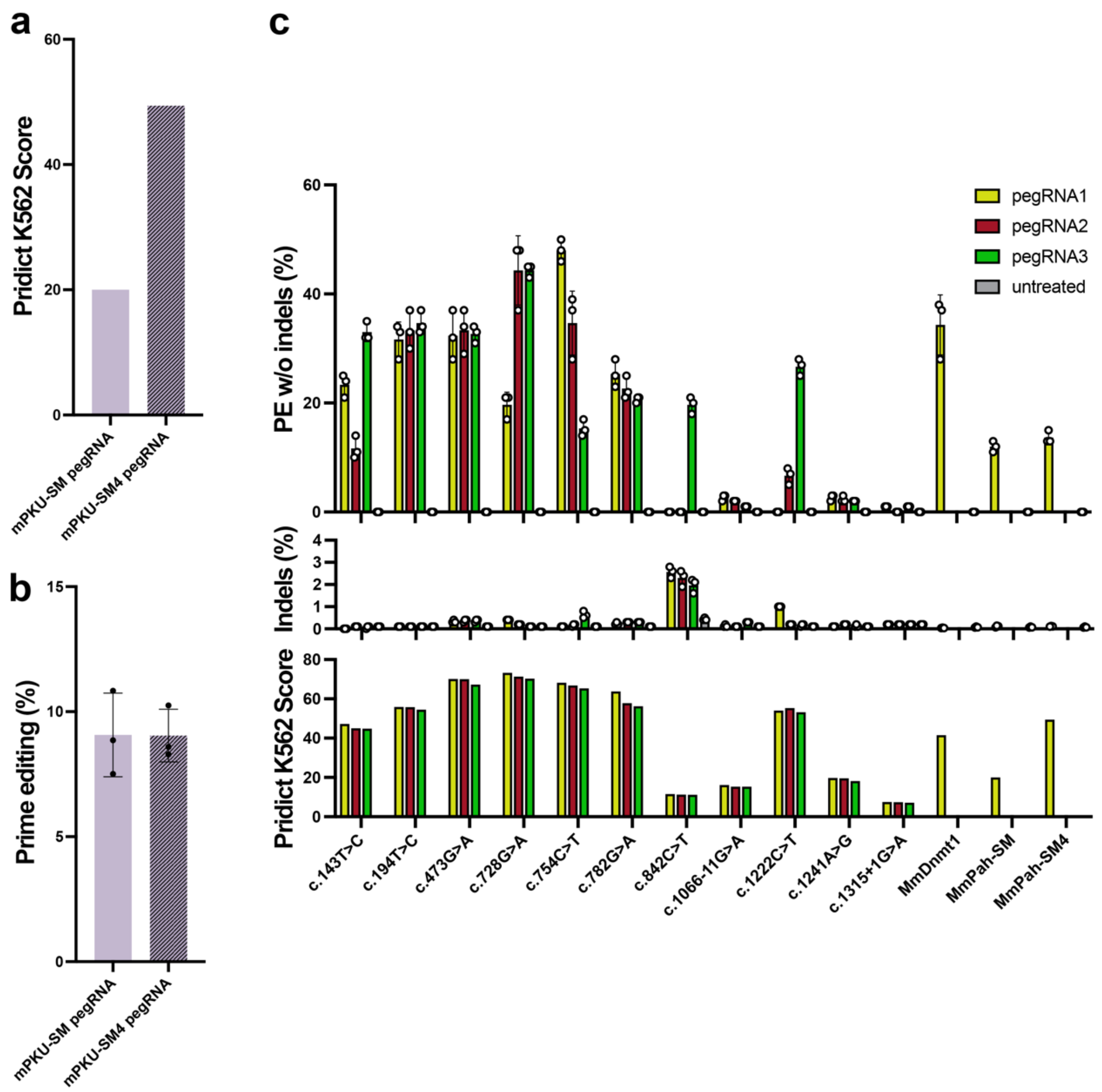

Figure S5 | Strategies to improve gene editing efficiency

(**a**) Use of Pridict2.0 for optimization of pegRNAs targeting the mouse *Pah*^{enu2} locus sequence with or without silent bystander mutations. **(b)** The activity of pegRNAs from **(a)** in K562 reporter cell line. Each value represents mean of n=3 individual biological replicates. (**c**) Prime editing rates (upper panel) and indel rates (middle panel) in a K562 reporter cell line harboring the most common human pathogenic PKU mutations and the pathogenic mouse *Pah*^{enu2} and *Dnmt1* locus as a comparison. Three pegRNAs were designed for each locus and optimized by adding silent bystander mutations and predicting their activity with Pridict 2.0 (lower panel). Values represent mean +/- s.d. of n=3 independent biological replicates.

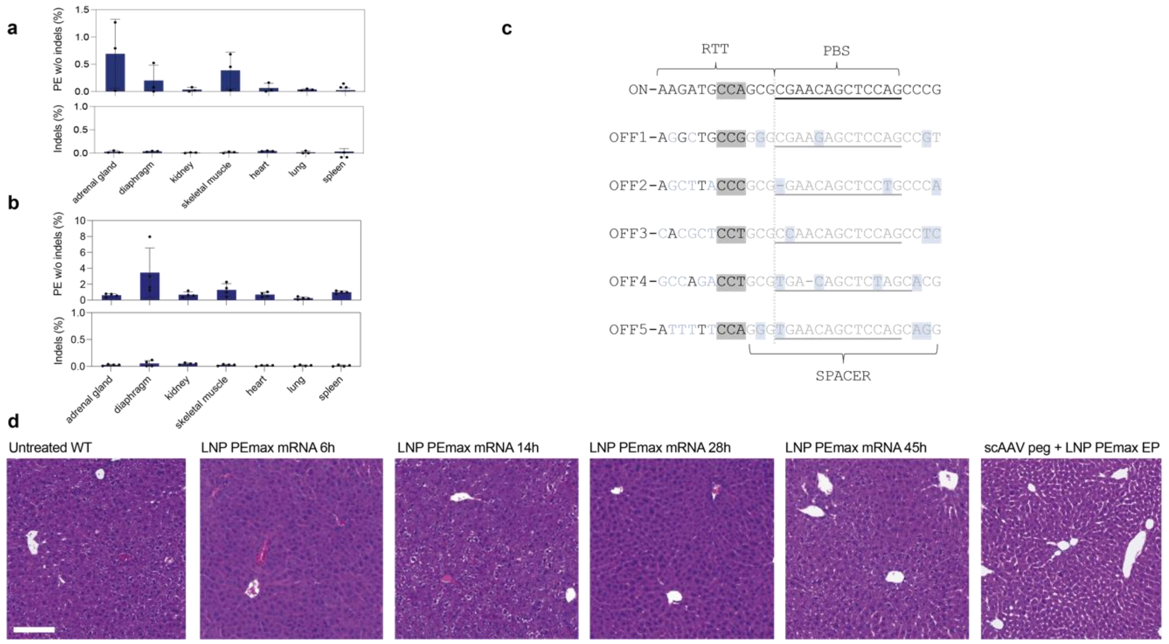

Figure S6 | Editing rates in non-liver tissues and liver histology at different timepoints after mRNA-LNP delivery. (**a, b**) G-to-C conversion rates (upper panel) and indel rates (lower panel) at the *Dnmt1* locus on genomic DNA isolated from tissues other than the liver from animals pretreated with scAAV encoding for the tevopreQ_1-modified pegRNA targeting *Dnmt1* and dosed once with 3 mg/kg either PE2 or PEmax mRNA (n=3) (a) or with PE7 mRNA-LNP and pegRNA-LNP (n=3) (b). Editing rates were assessed by NGS. (**c**) Top five off-target sites of the *Dnmt1*-targeting tevopreQ_1-modified pegRNA identified by CHANGE-seq[16]. Indicated are the spacer, the primer binding site (underlined, PBS) and reverse transcriptase template (RTT) of the target site (ON) (black) and off-target sites (OFF1-OFF5) (spacer; gray letters). Mismatches to the target site are in light blue (letters; mismatches to RTT, highlighted letters; mismatches to spacer). The bases complementary to the PAM site are highlighted in grey. The site of the DNA nick is indicated by a dotted line. (**d**) Histological images of hematoxylin and eosin (H&E) stained 5 µm thick liver tissue. Images are representative of two individual animals. Scale bar: 100 µm.

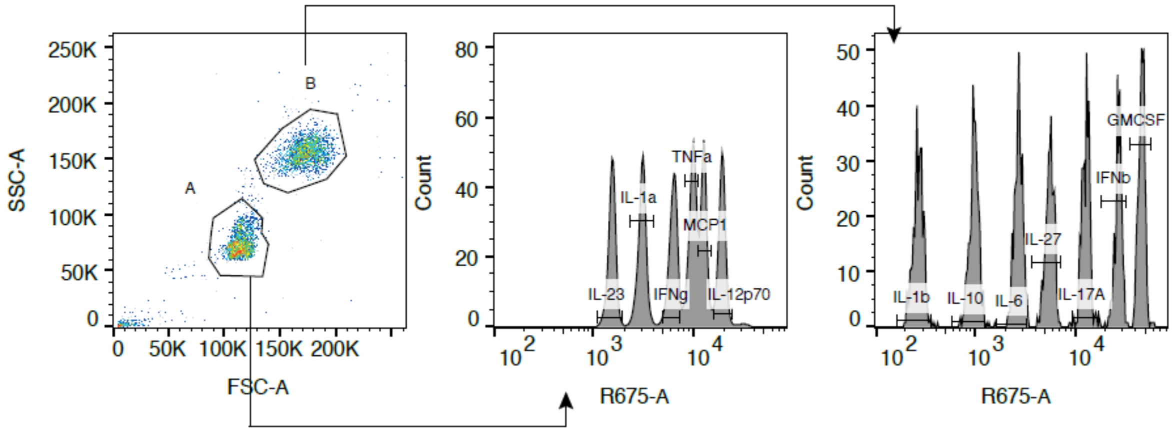

Figure S7 | Gating strategy identifying proinflammatory markers.

Proinflammatory markers were defined by flow cytometry.

Supplementary Note 1 | Amino acid sequences of AAV vectors used in this study.

N-int-PEmax (1-712): BPSV40NLS – Cas9 (1-712) – (GGGGS)3 linker – Npu N-intein – SV40-NLS

MKRTADGSEFESPKKKRKVDKKYSIGLDIGTNSVGWAVITDEYKVPSKKFKVLGNTDRHSIKKNLIGALLFDSG
ETAEATRLKRTARRRYTRRKNRICYLQEIFSNEMAKVDDSFFHRLEESFLVEEDKKHERHPIFGNIVDEVAYHEK
YPTIYHLRKKLVDSTDKADLRLIYLALAHMIKFRGHFLIEGDLNPDNSDVDKLFIQLVQTYNQLFEENPINASGV
DAKAILSARLSKSRKLENLIAQLPGEKKNGLFGNLIALSLGLTPNFKSNFDLAEDAKLQLSKDTYDDDLDNLLAQ
IGDQYADLFLAAKNLSDAILLSDILRVNTEITKAPLSASMIKRYDEHHQDLTLLKALVRQQLPEKYKEIFFDQSKN
GYAGYIDGGASQEEFYKFIKPILEKMDGTEELLVKLKREDLLRKQRTFDNGSIPHQIHLGELHAILRRQEDFYPFL
KDNREKIEKILTFRIPYYVGPLARGNSRFAWMTRKSEETITPWNFEEVVDKGASAQSFIERMTNFDKNLPNEKVL
PKHSLLYEYFTVYNELTKVKYVTEGMRKPAFLSGEQKKAIVDLLFKTNRKVTVKQLKEDYFKKIECFDSVEISG
VEDRFNASLGTYHDLLKIIKDKDFLDNEENEDILEDIVLTLTLFEDREMIEERLKTYAHLFDDKVMKQLKRRRYT
GWGRLSRKLINGIRDKQSGKTILDFLKSDGFANRNFMQLIHDDSLTFKEDIQKAQVGGGGSGGGGSGGGGSCLS
YETEILTVEYGLLPIGKIVEKRIECTVYSVDNNGNIYTQPVAQWHDRGEQEVFEYCLEDGSLIRATKDHKFMTVD
GQMLPIDEIFERELDLMRVDNLPNEFEPKKKRKV*

C-int-PEmax (713-1368): BPSV40NLS – Npu C-intein – (GGGGS)3 linker – Cas9 (713-1368) – (SGGS)2 – BPSV40NLS – (SGGS)2 – M-MLV-RT-dRnH – SGGS – BPSV40NLS – cMyc NLS

MKRTADGSEFESPKKKRKVIKIATRKYLGKQNVYDIGVERDHNFALKNGFIASNSGGGGSGGGGSGGGGSSGQ
GDSLHEHIANLAGSPAIKKGILQTVKVVDELVKVMGRHKPENIVIEMARENQTTQKGQKNSRERMKRIEEGIKE
LGSQILKEHPVENTQLQNEKLYLYYLQNGRDMYVDQELDINRLSDYDVDAIVPQSFLKDDSIDNKVLTRSDKNR
GKSDNVPSEEVVKKMKNYWRQLLNAKLITQRKFDNLTKAERGGLSELDKAGFIKRQLVETRQITKHVAQILDSR
MNTKYDENDKLIREVKVITLKSKLVSDFRKDFQFYKVREINNYHHAHDAYLNAVVGTALIKKYPKLESEFVYG
DYKVYDVRKMIAKSEQEIGKATAKYFFYSNIMNFFKTEITLANGEIRKRPLIETNGETGEIVWDKGRDFATVRKV
LSMPQVNIVKKTEVQTGGFSKESILPKRNSDKLIARKKDWDPKKYGGFDSPTVAYSVLVVAKVEKGKSKKLKS
VKELLGITIMERSSFEKNPIDFLEAKGYKEVKKDLIIKLPKYSLFELENGRKRMLASAGELQKGNELALPSKYVNF
LYLASHYEKLKGSPEDNEQKQLFVEQHKHYLDEIIEQISEFSKRVILADANLDKVLSAYNKHRDKPIREQAENIIH
LFTLTNLGAPAAFKYFDTTIDRKRYTSTKEVLDATLIHQSITGLYETRIDLSQLGGDSGGSSGGSKRTADGSEFES
PKKKRKVSGGSSGGSTLNIEDEYRLHETSKEPDVSLGSTWLSDFPQAWAETGGMGLAVRQAPLIIPLKATSTPVS
IKQYPMSQEARLGIKPHIQRLLDQGILVPCQSPWNTPLLPVKKPGTNDYRPVQDLREVNKRVEDIHPTVPNPYNL
LSGLPPSHQWYTVLDLKDAFFCLRLHPTSQPLFAFEWRDPEMGISGQLTWTRLPQGFKNSPTLFNEALHRDLAD
FRIQHPDLILLQYVDDLLLAATSELDCQQGTRALLQTLGNLGYRASAKKAQICQKQVKYLGYLLKEGQRWLTE
ARKETVMGQPTPKTPRQLREFLGKAGFCRLFIPGFAEMAAPLYPLTKPGTLFNWGPDQQKAYQEIKQALLTAPA
LGLPDLTKPFELFVDEKQGYAKGVLTQKLGPWRRPVAYLSKKLDPVAAGWPPCLRMVAAIAVLTKDAGKLTM
GQPLVILAPHAVEALVKQPPDRWLSNARMTHYQALLLDTDRVQFGPVVALNPSGGSKRTADGSEFESPKKKRK
VGSGPAAKRVKLD*

N-int-PEmax (1-1153): BPSV40NLS – Cas9 (1-712) – (GGGGS)3 linker – Npu N-intein – SV40-NLS

MKRTADGSEFESPKKKRKVDKKYSIGLDIGTNSVGWAVITDEYKVPSKKFKVLGNTDRHSIKKNLIGALLFDSG
ETAEATRLKRTARRRYTRRKNRICYLQEIFSNEMAKVDDSFFHRLEESFLVEEDKKHERHPIFGNIVDEVAYHEK
YPTIYHLRKKLVDSTDKADLRLIYLALAHMIKFRGHFLIEGDLNPDNSDVDKLFIQLVQTYNQLFEENPINASGV
DAKAILSARLSKSRKLENLIAQLPGEKKNGLFGNLIALSLGLTPNFKSNFDLAEDAKLQLSKDTYDDDLDNLLAQ
IGDQYADLFLAAKNLSDAILLSDILRVNTEITKAPLSASMIKRYDEHHQDLTLLKALVRQQLPEKYKEIFFDQSKN
GYAGYIDGGASQEEFYKFIKPILEKMDGTEELLVKLKREDLLRKQRTFDNGSIPHQIHLGELHAILRRQEDFYPFL
KDNREKIEKILTFRIPYYVGPLARGNSRFAWMTRKSEETITPWNFEEVVDKGASAQSFIERMTNFDKNLPNEKVL
PKHSLLYEYFTVYNELTKVKYVTEGMRKPAFLSGEQKKAIVDLLFKTNRKVTVKQLKEDYFKKIECFDSVEISG
VEDRFNASLGTYHDLLKIIKDKDFLDNEENEDILEDIVLTLTLFEDREMIEERLKTYAHLFDDKVMKQLKRRRYT
GWGRLSRKLINGIRDKQSGKTILDFLKSDGFANRNFMQLIHDDSLTFKEDIQKAQVSGQGDSLHEHIANLAGSPA
IKKGILQTVKVVDELVKVMGRHKPENIVIEMARENQTTQKGQKNSRERMKRIEEGIKELGSQILKEHPVENTQLQ
NEKLYLYYLQNGRDMYVDQELDINRLSDYDVDAIVPQSFLKDDSIDNKVLTRSDKNRGKSDNVPSEEVVKKMK
NYWRQLLNAKLITQRKFDNLTKAERGGLSELDKAGFIKRQLVETRQITKHVAQILDSRMNTKYDENDKLIREVK
VITLKSKLVSDFRKDFQFYKVREINNYHHAHDAYLNAVVGTALIKKYPKLESEFVYGDYKVYDVRKMIAKSEQ
EIGKATAKYFFYSNIMNFFKTEITLANGEIRKRPLIETNGETGEIVWDKGRDFATVRKVLSMPQVNIVKKTEVQT
GGFSKESILPKRNSDKLIARKKDWDPKKYGGFDSPTVAYSVLVVAKVEKGKGGGGSGGGGSGGGGSCLSYETE
ILTVEYGLLPIGKIVEKRIECTVYSVDNNGNIYTQPVAQWHDRGEQEVFEYCLEDGSLIRATKDHKFMTVDGQM
LPIDEIFERELDLMRVDNLPN*

C-int-PEmax (1154-1368): BPSV40NLS – Npu C-intein – (GGGGS)3 linker – Cas9 (713-1368) – (SGGS)2 – BPSV40NLS – (SGGS)2 – M-MLV-RT-dRnH – SGGS – BPSV40NLS – cMyc NLS

MKRTADGSEFESPKKKRKVIKIATRKYLGKQNVYDIGVERDHNFALKNGFIASNSGGGGSGGGGSGGGGSSKK
LKSVKELLGITIMERSSFEKNPIDFLEAKGYKEVKKDLIIKLPKYSLFELENGRKRMLASAGELQKGNELALPSKY

VNFLYLASHYEKLKGSPEDNEQKQLFVEQHKHYLDEIIEQISEFSKRVILADANLDKVLSAYNKHRDKPIREQAE<br>NIIHLFTLTNLGAPAAFKYFDTTIDRKRYTSTKEVLDATLIHQSITGLYETRIDLSQLGGDSGGSSGGSKRTADGSE<br>FESPKKKRKVSGGSSGGSTLNIEDEYRLHETSKEPDVSLGSTWLSDFPQAWAETGGMGLAVRQAPLIIPLKATST<br>PVSIKQYPMSQEARLGIKPHIQRLLDQGILVPCQSPWNTPLLPVKKPGTNDYRPVQDLREVNKRVEDIHPTVPNP<br>YNLLSGLPPSHQWYTVLDLKDAFFCLRLHPTSQPLFAFEWRDPEMGISGQLTWTRLPQGFKNSPTLFNEALHRD<br>LADFRIQHPDLILLQYVDDLLLAATSELDCQQGTRALLQTLGNLGYRASAKKAQICQKQVKYLGYLLKEGQRW<br>LTEARKETVMGQPTPKTPRQLREFLGKAGFCRLFIPGFAEMAAPLYPLTKPGTLFNWGPDQQKAYQEIKQALLT<br>APALGLPDLTKPFELFVDEKQGYAKGVLTQKLGPWRRPVAYLSKKLDPVAAGWPPCLRMVAAIAVLTKDAGK<br>LTMGQPLVILAPHAVEALVKQPPDRWLSNARMTHYQALLLDTDRVQFGPVVALNPSGGSKRTADGSEFESPKK<br>KRKVGSGPAAKRVKLD*
**tagRFP**
MVSKGEELIKENMHMKLYMEGTVNNHHFKCTSEGEGKPYEGTQTMRIKVVEGGPLPFAFDILATSFMYGSRTFI<br>NHTQGIPDFFKQSFPEGFTWERVTTYEDGGVLTATQDTSLQDGCLIYNVKIRGVNFPSNGPVMQKKTLGWEANT<br>EMLYPADGGLEGRSDMALKLVGGGHLICNFKTTYRSKKPAKNLKMPGVYYVDHRLERIKEADKETYVEQHEV<br>AVARYCDLPSKLGHKLN*
**PE2 mRNA:** BPSV40NLS – Cas9 (H840A; PE2) – (SGGS)2 – XTEN – (SGGS)2 – M-MLV-RT (PE2) – SGGS –<br>BPSV40NLS
MKRTADGSEFESPKKKRKVDKKYSIGLDIGTNSVGWAVITDEYKVPSKKFKVLGNTDRHSIKKNLIGALLFDSG<br>ETAEATRLKRTARRRYTRRKNRICYLQEIFSNEMAKVDDSFFHRLEESFLVEEDKKHERHPIFGNIVDEVAYHEK<br>YPTIYHLRKKLVDSTDKADLRLIYLALAHMIKFRGHFLIEGDLNPDNSDVDKLFIQLVQTYNQLFEENPINASGV<br>DAKAILSARLSKSRRLENLIAQLPGEKKNGLFGNLIALSLGLTPNFKSNFDLAEDAKLQLSKDTYDDDLDNLLAQ<br>IGDQYADLFLAAKNLSDAILLSDILRVNTEITKAPLSASMIKRYDEHHQDLTLLKALVRQQLPEKYKEIFFDQSKN<br>GYAGYIDGGASQEEFYKFIKPILEKMDGTEELLVKLNREDLLRKQRTFDNGSIPHQIHLGELHAILRRQEDFYPFL<br>KDNREKIEKILTFRIPYYVGPLARGNSRFAWMTRKSEETITPWNFEEVVDKGASAQSFIERMTNFDKNLPNEKVL<br>PKHSLLYEYFTVYNELTKVKYVTEGMRKPAFLSGEQKKAIVDLLFKTNRKVTVKQLKEDYFKKIECFDSVEISG<br>VEDRFNASLGTYHDLLKIIKDKDFLDNEENEDILEDIVLTLTLFEDREMIEERLKTYAHLFDDKVMKQLKRRRYT<br>GWGRLSRKLINGIRDKQSGKTILDFLKSDGFANRNFMQLIHDDSLTFKEDIQKAQVSGQGDSLHEHIANLAGSPA<br>IKKGILQTVKVVDELVKVMGRHKPENIVIEMARENQTTQKGQKNSRERMKRIEEGIKELGSQILKEHPVENTQLQ<br>NEKLYLYYLQNGRDMYVDQELDINRLSDYDVDAIVPQSFLKDDSIDNKVLTRSDKNRGKSDNVPSEEVVKKMK<br>NYWRQLLNAKLITQRKFDNLTKAERGGLSELDKAGFIKRQLVETRQITKHVAQILDSRMNTKYDENDKLIREVK<br>VITLKSKLVSDFRKDFQFYKVREINNYHHAHDAYLNAVVGTALIKKYPKLESEFVYGDYKVYDVRKMIAKSEQ<br>EIGKATAKYFFYSNIMNFFKTEITLANGEIRKRPLIETNGETGEIVWDKGRDFATVRKVLSMPQVNIVKKTEVQT<br>GGFSKESILPKRNSDKLIARKKDWDPKKYGGFDSPTVAYSVLVVAKVEKGKSKKLKSVKELLGITIMERSSFEK<br>NPIDFLEAKGYKEVKKDLIIKLPKYSLFELENGRKRMLASAGELQKGNELALPSKYVNFLYLASHYEKLKGSPED<br>NEQKQLFVEQHKHYLDEIIEQISEFSKRVILADANLDKVLSAYNKHRDKPIREQAENIIHLFTLTNLGAPAAFKYF<br>DTTIDRKRYTSTKEVLDATLIHQSITGLYETRIDLSQLGGDSGGSSGGSSGSETPGTSESATPESSGGSSGGSSTLNI<br>EDEYRLHETSKEPDVSLGSTWLSDFPQAWAETGGMGLAVRQAPLIIPLKATSTPVSIKQYPMSQEARLGIKPHIQ<br>RLLDQGILVPCQSPWNTPLLPVKKPGTNDYRPVQDLREVNKRVEDIHPTVPNPYNLLSGLPPSHQWYTVLDLKD<br>AFFCLRLHPTSQPLFAFEWRDPEMGISGQLTWTRLPQGFKNSPTLFNEALHRDLADFRIQHPDLILLQYVDDLLL<br>AATSELDCQQGTRALLQTLGNLGYRASAKKAQICQKQVKYLGYLLKEGQRWLTEARKETVMGQPTPKTPRQL<br>REFLGKAGFCRLFIPGFAEMAAPLYPLTKPGTLFNWGPDQQKAYQEIKQALLTAPALGLPDLTKPFELFVDEKQ<br>GYAKGVLTQKLGPWRRPVAYLSKKLDPVAAGWPPCLRMVAAIAVLTKDAGKLTMGQPLVILAPHAVEALVKQ<br>PPDRWLSNARMTHYQALLLDTDRVQFGPVVALNPATLLPLPEEGLQHNCLDILAEAHGTRPDLTDQPLPDADHT<br>WYTDGSSLLQEGQRKAGAAVTTETEVIWAKALPAGTSAQRAELIALTQALKMAEGKKLNVYTDSRYAFATAHI<br>HGEIYRRRGWLTSEGKEIKNKDEILALLKALFLPKRLSIIHCPGHQKGHSAEARGNRMADQAARKAAITETPDTS<br>TLLIENSSPSGGSKRTADGSEFEPKKKRKV*
**PEmax mRNA:** BPSV40NLS – Cas9 (H840A; PEmax) – (SGGS)2 – BPSV40NLS – (SGGS)2 – M-MLV-RT (PEmax)<br>– SGGS – BPSV40NLS – cMyc NLS
MKRTADGSEFESPKKKRKVDKKYSIGLDIGTNSVGWAVITDEYKVPSKKFKVLGNTDRHSIKKNLIGALLFDSG<br>ETAEATRLKRTARRRYTRRKNRICYLQEIFSNEMAKVDDSFFHRLEESFLVEEDKKHERHPIFGNIVDEVAYHEK<br>YPTIYHLRKKLVDSTDKADLRLIYLALAHMIKFRGHFLIEGDLNPDNSDVDKLFIQLVQTYNQLFEENPINASGV<br>DAKAILSARLSKSRKLENLIAQLPGEKKNGLFGNLIALSLGLTPNFKSNFDLAEDAKLQLSKDTYDDDLDNLLAQ<br>IGDQYADLFLAAKNLSDAILLSDILRVNTEITKAPLSASMIKRYDEHHQDLTLLKALVRQQLPEKYKEIFFDQSKN<br>GYAGYIDGGASQEEFYKFIKPILEKMDGTEELLVKLKREDLLRKQRTFDNGSIPHQIHLGELHAILRRQEDFYPFL<br>KDNREKIEKILTFRIPYYVGPLARGNSRFAWMTRKSEETITPWNFEEVVDKGASAQSFIERMTNFDKNLPNEKVL<br>PKHSLLYEYFTVYNELTKVKYVTEGMRKPAFLSGEQKKAIVDLLFKTNRKVTVKQLKEDYFKKIECFDSVEISG<br>VEDRFNASLGTYHDLLKIIKDKDFLDNEENEDILEDIVLTLTLFEDREMIEERLKTYAHLFDDKVMKQLKRRRYT<br>GWGRLSRKLINGIRDKQSGKTILDFLKSDGFANRNFMQLIHDDSLTFKEDIQKAQVSGQGDSLHEHIANLAGSPA

IKKGILQTVKVVDELVKVMGRHKPENIVIEMARENQTTQKGQKNSRERMKRIEEGIKELGSQILKEHPVENTQLQ
NEKLYLYYLQNGRDMYVDQELDINRLSDYDVDAIVPQSFLKDDSIDNKVLTRSDKNRGKSDNVPSEEVVKKMK
NYWRQLLNAKLITQRKFDNLTKAERGGLSELDKAGFIKRQLVETRQITKHVAQILDSRMNTKYDENDKLIREVK
VITLKSKLVSDFRKDFQFYKVREINNYHHAHDAYLNAVVGTALIKKYPKLESEFVYGDYKVYDVRKMIAKSEQ
EIGKATAKYFFYSNIMNFFKTEITLANGEIRKRPLIETNGETGEIVWDKGRDFATVRKVLSMPQVNIVKKTEVQT
GGFSKESILPKRNSDKLIARKKDWDPKKYGGFDSPTVAYSVLVVAKVEKGKSKKLKSVKELLGITIMERSSFEK
NPIDFLEAKGYKEVKKDLIIKLPKYSLFELENGRKRMLASAGELQKGNELALPSKYVNFLYLASHYEKLKGSPED
NEQKQLFVEQHKHYLDEIIEQISEFSKRVILADANLDKVLSAYNKHRDKPIREQAENIIHLFTLTNLGAPAAFKYF
DTTIDRKRYTSTKEVLDATLIHQSITGLYETRIDLSQLGGDSGGSSGGSKRTADGSEFESPKKKRKVSGGSSGGST
LNIEDEYRLHETSKEPDVSLGSTWLSDFPQAWAETGGMGLAVRQAPLIIPLKATSTPVSIKQYPMSQEARLGIKP
HIQRLLDQGILVPCQSPWNTPLLPVKKPGTNDYRPVQDLREVNKRVEDIHPTVPNPYNLLSGLPPSHQWYTVLD
LKDAFFCLRLHPTSQPLFAFEWRDPEMGISGQLTWTRLPQGFKNSPTLFNEALHRDLADFRIQHPDLILLQYVDD
LLLAATSELDCQQGTRALLQTLGNLGYRASAKKAQICQKQVKYLGYLLKEGQRWLTEARKETVMGQPTPKTP
RQLREFLGKAGFCRLFIPGFAEMAAPLYPLTKPGTLFNWGPDQQKAYQEIKQALLTAPALGLPDLTKPFELFVDE
KQGYAKGVLTQKLGPWRRPVAYLSKKLDPVAAGWPPCLRMVAAIAVLTKDAGKLTMGQPLVILAPHAVEALV
KQPPDRWLSNARMTHYQALLLDTDRVQFGPVVALNPATLLPLPEEGLQHNCLDILAEAHGTRPDLTDQPLPDA
DHTWYTDGSSLLQEGQRKAGAAVTTETEVIWAKALPAGTSAQRAELIALTQALKMAEGKKLNVYTDSRYAFA
TAHIHGEIYRRRGWLTSEGKEIKNKDEILALLKALFLPKRLSIIHCPGHQKGHSAEARGNRMADQAARKAAITET
PDTSTLLIENSSPSGGSKRTADGSEFESPKKKRKVGSGPAAKRVKLD*

PE7 mRNA: BPSV40NLS – Cas9 (H840A; PEmax) – (SGGS)2 – BPSV40NLS – (SGGS)2 – M-MLV-RT (PEmax) – La - SGGS – BPSV40NLS – cMyc NLS

MKRTADGSEFESPKKKRKVDKKYSIGLDIGTNSVGWAVITDEYKVPSKKFKVLGNTDRHSIKKNLIGALLFDSG
ETAEATRLKRTARRRYTRRKNRICYLQEIFSNEMAKVDDSFFHRLEESFLVEEDKKHERHPIFGNIVDEVAYHEK
YPTIYHLRKKLVDSTDKADLRLIYLALAHMIKFRGHFLIEGDLNPDNSDVDKLFIQLVQTYNQLFEENPINASGV
DAKAILSARLSKSRKLENLIAQLPGEKKNGLFGNLIALSLGLTPNFKSNFDLAEDAKLQLSKDTYDDDLDNLLAQ
IGDQYADLFLAAKNLSDAILLSDILRVNTEITKAPLSASMIKRYDEHHQDLTLLKALVRQQLPEKYKEIFFDQSKN
GYAGYIDGGASQEEFYKFIKPILEKMDGTEELLVKLKREDLLRKQRTFDNGSIPHQIHLGELHAILRRQEDFYPFL
KDNREKIEKILTFRIPYYVGPLARGNSRFAWMTRKSEETITPWNFEEVVDKGASAQSFIERMTNFDKNLPNEKVL
PKHSLLYEYFTVYNELTKVKYVTEGMRKPAFLSGEQKKAIVDLLFKTNRKVTVKQLKEDYFKKIECFDSVEISG
VEDRFNASLGTYHDLLKIIKDKDFLDNEENEDILEDIVLTLTLFEDREMIEERLKTYAHLFDDKVMKQLKRRRYT
GWGRLSRKLINGIRDKQSGKTILDFLKSDGFANRNFMQLIHDDSLTFKEDIQKAQVSGQGDSLHEHIANLAGSPA
IKKGILQTVKVVDELVKVMGRHKPENIVIEMARENQTTQKGQKNSRERMKRIEEGIKELGSQILKEHPVENTQLQ
NEKLYLYYLQNGRDMYVDQELDINRLSDYDVDAIVPQSFLKDDSIDNKVLTRSDKNRGKSDNVPSEEVVKKMK
NYWRQLLNAKLITQRKFDNLTKAERGGLSELDKAGFIKRQLVETRQITKHVAQILDSRMNTKYDENDKLIREVK
VITLKSKLVSDFRKDFQFYKVREINNYHHAHDAYLNAVVGTALIKKYPKLESEFVYGDYKVYDVRKMIAKSEQ
EIGKATAKYFFYSNIMNFFKTEITLANGEIRKRPLIETNGETGEIVWDKGRDFATVRKVLSMPQVNIVKKTEVQT
GGFSKESILPKRNSDKLIARKKDWDPKKYGGFDSPTVAYSVLVVAKVEKGKSKKLKSVKELLGITIMERSSFEK
NPIDFLEAKGYKEVKKDLIIKLPKYSLFELENGRKRMLASAGELQKGNELALPSKYVNFLYLASHYEKLKGSPED
NEQKQLFVEQHKHYLDEIIEQISEFSKRVILADANLDKVLSAYNKHRDKPIREQAENIIHLFTLTNLGAPAAFKYF
DTTIDRKRYTSTKEVLDATLIHQSITGLYETRIDLSQLGGDSGGSSGGSKRTADGSEFESPKKKRKVSGGSSGGST
LNIEDEYRLHETSKEPDVSLGSTWLSDFPQAWAETGGMGLAVRQAPLIIPLKATSTPVSIKQYPMSQEARLGIKP
HIQRLLDQGILVPCQSPWNTPLLPVKKPGTNDYRPVQDLREVNKRVEDIHPTVPNPYNLLSGLPPSHQWYTVLD
LKDAFFCLRLHPTSQPLFAFEWRDPEMGISGQLTWTRLPQGFKNSPTLFNEALHRDLADFRIQHPDLILLQYVDD
LLLAATSELDCQQGTRALLQTLGNLGYRASAKKAQICQKQVKYLGYLLKEGQRWLTEARKETVMGQPTPKTP
RQLREFLGKAGFCRLFIPGFAEMAAPLYPLTKPGTLFNWGPDQQKAYQEIKQALLTAPALGLPDLTKPFELFVDE
KQGYAKGVLTQKLGPWRRPVAYLSKKLDPVAAGWPPCLRMVAAIAVLTKDAGKLTMGQPLVILAPHAVEALV
KQPPDRWLSNARMTHYQALLLDTDRVQFGPVVALNPATLLPLPEEGLQHNCLDILAEAHGTRPDLTDQPLPDA
DHTWYTDGSSLLQEGQRKAGAAVTTETEVIWAKALPAGTSAQRAELIALTQALKMAEGKKLNVYTDSRYAFA
TAHIHGEIYRRRGWLTSEGKEIKNKDEILALLKALFLPKRLSIIHCPGHQKGHSAEARGNRMADQAARKAAITET
PDTSTLLIENSSPSGSETPGTSESATPESSGGSSGGSMAENGDNEKMAALEAKICHQIEYYFGDFNLPRDKFLKEQI
KLDEGWVPLEIMIKFNRLNRLTTDFNVIVEALSKSKAELMEISEDKTKIRRSPSKPLPEVTDEYKNDVKNRSVYIK
GFPTDATLDDIKEWLEDKGQVLNIQMRRTLHKAFKGSIFVVFDSIESAKKFVETPGQKYKETDLLILFKDDYFAK
KNEERKQNKSGGSKRTADGSEFESPKKKRKVGSGPAAKRVKLD*

Supplementary Note 2 | Complete image of Western blot.

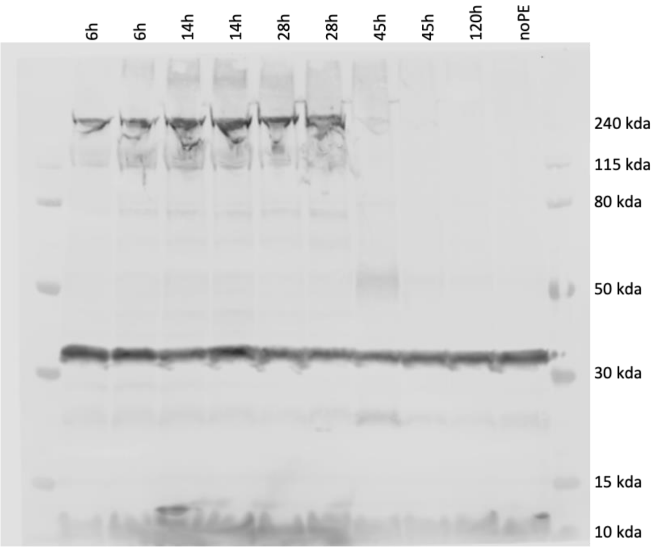

Supplementary Table 1 | pegRNA designs tested for correction of *Pah*^{enu2} mutations (5’ > 3’).

peg-mPKU-2.1	GCCTAATGTACTGTGTGCAG..scaffold..TTCCGGGTCTTCCACTGCACACAGTACA
peg-mPKU-2.1-tevo	GCCTAATGTACTGTGTGCAG..scaffold..TTCCGGGTCTTCCACTGCACACAGTACA-tevo
peg-mPKU-2.1-tmp	GCCTAATGTACTGTGTGCAG..scaffold..TTCCGGGTCTTCCACTGCACACAGTACA-tmp
peg-mPKU-SM_tevo	GCCTAATGTACTGTGTGCAG..scaffold..TTCCGAGTCTTTCACTGCACACAGTACA-tevo
peg-mPKU-SM4_tevo	GCCTAATGTACTGTGTGCAG..scaffold..TTCCGAGTGTTTCATTGCACACAGTACAT-tevo
peg-Dnmt1_tevo	GCGGGCTGGAGCTGTTCGCGC..scaffold..AAGATGGCAGCGCGAACAGCTCCAG-tevo
Hs_Pah_1222_peg1	GCTTTGCTGCCACAATACCT-scaffold-GAACTGAGAAGGGTCGTGGTATTGTGGCAGCAAA-tevo
Hs_Pah_1222_peg2	GCTTTGCTGCCACAATACCT-scaffold-GAACTGAGAAGGGTCGCGGTATTGTGGCAG-tevo
Hs_Pah_1222_peg3	GCTTTGCTGCCACAATACCT-scaffold-GAACTGAGAAGGGGCGTGGTATTGTGGCAGCAAA-tevo
Hs_Pah_1066_peg1	GCTATCCTTGGTTCCTGTGA-scaffold-TTACAGGTATGACCTTCACAGGAACCAAGGA-tevo
Hs_Pah_1066_peg2	GCTATCCTTGGTTCCTGTGA-scaffold-GTGAATTACAGGTATGACCGTCACAGGAACCAAGGA-tevo
Hs_Pah_1066_peg3	GCTGGGCTCCTGTCATCCTT-scaffold-GAAGGTCATACCTGGAGTTCACCAAAGGATGACAGGAGCCCA-tevo
Hs_Pah_782_peg1	GCTGTGTGCAGTGGAAGACT-scaffold-TGGCCTTTAGGGTCTTCCACTGCACAC-tevo
Hs_Pah_782_peg2	GCTGTGTGCAGTGGAAGACT-scaffold-TGGCCTTCAGGGTCTTCCACTGCACACA-tevo
Hs_Pah_782_peg3	GCTGTGTGCAGTGGAAGACT-scaffold-GCCTGGCGTTTAGGGTCTTCCACTGCACAC-tevo
Hs_Pah_728_peg1	GCAGGCCAGCCACAGGTTGG-scaffold-TGGTTTCCGGCTGCGACCTGTGGCTGG-tevo
Hs_Pah_728_peg2	GCAGGCCAGCCACAGGTTGG-scaffold-TGGTTTCCGGTTGCGACCTGTGGCTGG-tevo
Hs_Pah_728_peg3	GAAGCAGGCCAGCCACAGGT-scaffold-TGGTTTCCGCTTGAGGCCTGTGGCTGGCCT-tevo
Hs_Pah_1315_peg1	GGAAACCGAGTGGCCTCGTA-scaffold-ATTAACAGTAAGTAATTTACACCTTACGAGGCCACTCGGTT-tevo

Hs_Pah_1315_peg2	GACCCAGCAGCTTAAGATTT-scaffold-GTAAGGTGTAAATTACTTACTGTTGATCGAATCAGCCAAAATCTTAAGCTGCTGGG-tevo
Hs_Pah_1315_peg3	GGAAACCGAGTGGCCTCGTA-scaffold-ATTAACAGTAAGTAATTTACACCTTACGAGGCCACTCGGTTT-tevo
Hs_Pah_842_peg1	GCCTGGCAACTGGTAGCTGG-scaffold-CCATGTATACCCCCGAACCGTGAGTACTCTCCTCCAGCTACCAGTTGCCAG-tevo
Hs_Pah_842_peg2	GCCTGGCAACTGGTAGCTGG-scaffold-CCATGTATACCCCCGAACCGTGAGTACTATCCTCCAGCTACCAGTTGCCAG-tevo
Hs_Pah_842_peg3	GCCTGGCAACTGGTAGCTGG-scaffold-CGAACCGTGAGTACTGTCCTCCAGCTACCAGTT-tevo
Hs_Pah_473_peg1	GTCCTGTGTACCGTGCAAAA-scaffold-AAACTGCTTGCGGCGTGCACGGTACACAGG-tevo
Hs_Pah_473_peg2	GTCCTGTGTACCGTGCAAAA-scaffold-AAACTGCTTGCGTCGTGCACGGTACACAGG-tevo
Hs_Pah_473_peg3	GTCCTGTGTACCGTGCAAAA-scaffold-AAACTGCTTGCGTCGTGCACGGTACACAG-tevo
Hs_Pah_1241_peg1	GCCTCAATCCTTTGGGTGTA-scaffold-CCTTCTCAGTTCGCTACGATCCGTACACCCAAAGGATTGAG-tevo
Hs_Pah_1241_peg2	GCCTCAATCCTTTGGGTGTA-scaffold-TTCTCAGTTCGCTACGATCCGTACACCCAAAGGATTGAG-tevo
Hs_Pah_1241_peg3	GCCTCAATCCTTTGGGTGTA-scaffold-AGTTCGCTACGATCCATACACCCAAAGGATTGAG-tevo
Hs_Pah_143_peg1	GAAAGAAGAAGTTGGTGCAT-scaffold-AATACTTTGGCAAGAGCACCAACTTCTT-tevo
Hs_Pah_143_peg2	GAAAGAAGAAGTTGGTGCAT-scaffold-AATACTTTGGCGAGTGCACCAACTTCTT-tevo
Hs_Pah_143_peg3	GAAAGAAGAAGTTGGTGCAT-scaffold-AATACTTTCGCAAGTGCACCAACTTCTT-tevo
Hs_Pah_194_peg1	GAGAAGGTCTAGATTCAGTG-scaffold-GTAAACCTGACGCATATTGAATCTAGACCTT-tevo
Hs_Pah_194_peg2	GAGAAGGTCTAGATTCAGTG-scaffold-ATGTAAACCTGACGCATATTGAATCTAGACCTT-tevo
Hs_Pah_194_peg3	GAGAAGGTCTAGATTCAGTG-scaffold-ATGTAAACCTGACGCATATTGAATCTAGACCT-tevo
Hs_Pah_754_peg1	GCCACCCAAGAAATCCCAAG-scaffold-TGGCCTGCTTAGTAGTAGGGATTTCTTGGGTG-tevo
Hs_Pah_754_peg2	GCCACCCAAGAAATCCCAAG-scaffold-TGGCCTGCTTAGTAGTCGGGATTTCTTGGGTG-tevo
Hs_Pah_754_peg3	GCCACCCAAGAAATCCCAAG-scaffold-TGCTTTCGAGTCGGGATTTCTTGGG-tevo

Supplementary Table 2 | Oligonucleotides used for this study.

HTS_PKU_fw	CTTTCCCTACACGACGCTCTTCCGATCTNNNNNNCCGTCCTGTTGCTGGCTTAC
HTS_PKU_rv	GGAGTTCAGACGTGTGCTCTTCCGATCTNNNNNNTGAGCATCCATTGTGGTTGG
peg-PKU_spacer_fw	ATGGTCTCGCACCGCCTAATGTACTGTGTGCAGGTTTCAGAGCTATGCTGGAAACAGC
peg-PKU-0SM_tevo_rv	ATGGTCTCGCGCGTGTACTGTGTGCAGTGGAAGACTCGGAAGCACCGACTCGGTGCCAC
peg-PKU-SM_tevo_rv	ATGGTCTCGCGCGTGTACTGTGTGCAGTGAAAGACTCGGAAGCACCGACTCGGTGCCAC
peg-PKU-SM4_tevo_rv	ATGGTCTCGCGCGATGTACTGTGTGCAATGAAACACTCGGAAGCACCGACTCGGTGCCAC
Hs_Pah_1222_peg1-3_spacer-fw	ATGGTCTCGCACCGCTTTGCTGCCACAATACCTGTTTCAGAGCTATGCTGGAAACAGC
Hs_Pah_1066_peg1_peg1-2_spacer-fw	ATGGTCTCGCACCGCTATCCTTGGTTCCTGTGAGTTTCAGAGCTATGCTGGAAACAGC
Hs_Pah_1066_peg3_spacer-fw	ATGGTCTCGCACCGCTGGGCTCCTGTCATCCTTGTTTCAGAGCTATGCTGGAAACAGC

Hs_Pah_782_peg1-3_spacer-fw	ATGGTCTCGCACCGCTGTGTGCAGTGGAAGACTGTTTCAGAGCTATGCTGGAAACAGC
Hs_Pah_728_peg1_peg1-2_spacer-fw	ATGGTCTCGCACCGCAGGCCAGCCACAGGTTGGGTTTCAGAGCTATGCTGGAAACAGC
Hs_Pah_728_peg3_spacer-fw	ATGGTCTCGCACCGAAGCAGGCCAGCCACAGGTGTTTCAGAGCTATGCTGGAAACAGC
Hs_Pah_1315_peg1_spacer-fw	ATGGTCTCGCACCGGAAACCGAGTGGCCTCGTAGTTTCAGAGCTATGCTGGAAACAGC
Hs_Pah_1315_peg2_peg2-3_spacer-fw	ATGGTCTCGCACCGACCCAGCAGCTTAAGATTTGTTTCAGAGCTATGCTGGAAACAGC
Hs_Pah_842_peg1-3_spacer-fw	ATGGTCTCGCACCGCCTGGCAACTGGTAGCTGGGTTTCAGAGCTATGCTGGAAACAGC
Hs_Pah_473_peg1-3_spacer-fw	ATGGTCTCGCACCGTCCTGTGTACCGTGCAAAAGTTTCAGAGCTATGCTGGAAACAGC
Hs_Pah_1241_peg1-3_spacer-fw	ATGGTCTCGCACCGCCTCAATCCTTTGGGTGTAGTTTCAGAGCTATGCTGGAAACAGC
Hs_Pah_143_peg1-3_spacer-fw	ATGGTCTCGCACCGAAAGAAGAAGTTGGTGCATGTTTCAGAGCTATGCTGGAAACAGC
Hs_Pah_194_peg1-3_spacer-fw	ATGGTCTCGCACCGAGAAGGTCTAGATTCAGTGGTTTCAGAGCTATGCTGGAAACAGC
Hs_Pah_754_peg1-3_spacer-fw	ATGGTCTCGCACCGCCACCCAAGAAATCCCAAGGTTTCAGAGCTATGCTGGAAACAGC
Hs_Pah_1222_peg1_tevo-rv	ATGGTCTCGCGCGTTTGCTGCCACAATACCACGACCCTTCTCAGTTCGCACCGACTCGGTGCCAC
Hs_Pah_1222_peg2_tevo-rv	ATGGTCTCGCGCGCTGCCACAATACCGCGACCCTTCTCAGTTCGCACCGACTCGGTGCCAC
Hs_Pah_1222_peg3_tevo-rv	ATGGTCTCGCGCGTTTGCTGCCACAATACCACGCCCCTTCTCAGTTCGCACCGACTCGGTGCCAC
Hs_Pah_1066_peg1_tevo-rv	ATGGTCTCGCGCGTCCTTGGTTCCTGTGAAGGTCATACCTGTAAGCACCGACTCGGTGCCAC
Hs_Pah_1066_peg2_tevo-rv	ATGGTCTCGCGCGTCCTTGGTTCCTGTGACGGTCATACCTGTAATTCACGCACCGACTCGGTGCCAC
Hs_Pah_1066_peg3_tevo-rv	ATGGTCTCGCGCGTGGGCTCCTGTCATCCTTTGGTGAACTCCAGGTATGACCTTCGCACCGACTCGGTGCCAC
Hs_Pah_782_peg1_tevo-rv	ATGGTCTCGCGCGGTGTGCAGTGGAAGACCCTAAAGGCCAGCACCGACTCGGTGCCAC
Hs_Pah_782_peg2_tevo-rv	ATGGTCTCGCGCGTGTGTGCAGTGGAAGACCCTGAAGGCCAGCACCGACTCGGTGCCAC
Hs_Pah_782_peg3_tevo-rv	ATGGTCTCGCGCGGTGTGCAGTGGAAGACCCTAAACGCCAGGCGCACCGACTCGGTGCCAC
Hs_Pah_728_peg1_tevo-rv	ATGGTCTCGCGCGCCAGCCACAGGTCGCAGCCGGAAACCAGCACCGACTCGGTGCCAC
Hs_Pah_728_peg2_tevo-rv	ATGGTCTCGCGCGCCAGCCACAGGTCGCAACCGGAAACCAGCACCGACTCGGTGCCAC
Hs_Pah_728_peg3_tevo-rv	ATGGTCTCGCGCGAGGCCAGCCACAGGCCTCAAGCGGAAACCAGCACCGACTCGGTGCCAC
Hs_Pah_1315_peg1_tevo-rv	ATGGTCTCGCGCGAACCGAGTGGCCTCGTAAGGTGTAAATTACTTACTGTTAATGCACCGACTCGGTGCCAC
Hs_Pah_1315_peg2_tevo-rv	ATGGTCTCGCGCGCCCAGCAGCTTAAGATTTTGGCTGATTCGATCAACAGTAAGTAATTTACACCTTACGCACCGACTCGGTGCCAC
Hs_Pah_1315_peg3_tevo-rv	ATGGTCTCGCGCGAAACCGAGTGGCCTCGTAAGGTGTAAATTACTTACTGTTAATGCACCGACTCGGTGCCAC
Hs_Pah_842_peg1_tevo-rv	ATGGTCTCGCGCGCTGGCAACTGGTAGCTGGAGGAGAGTACTCACGGTTCGGGGGTATACATGGGCACCGACTCGGTGCCAC
Hs_Pah_842_peg2_tevo-rv	ATGGTCTCGCGCGCTGGCAACTGGTAGCTGGAGGATAGTACTCACGGTTCGGGGGTATACATGGGCACCGACTCGGTGCCAC
Hs_Pah_842_peg3_tevo-rv	ATGGTCTCGCGCGAACTGGTAGCTGGAGGACAGTACTCACGGTTCGGCACCGACTCGGTGCCAC

Hs_Pah_473_peg1_tevo-rv	ATGGTCTCGCGCGCCTGTGTACCGTGCACGCCGCAAGCAGTTTGCACCGACTCGGTGCCAC
Hs_Pah_473_peg2_tevo-rv	ATGGTCTCGCGCGCCTGTGTACCGTGCACGACGCAAGCAGTTTGCACCGACTCGGTGCCAC
Hs_Pah_473_peg3_tevo-rv	ATGGTCTCGCGCGCTGTGTACCGTGCACGACGCAAGCAGTTTGCACCGACTCGGTGCCAC
Hs_Pah_1241_peg1_tevo-rv	ATGGTCTCGCGCGCTCAATCCTTTGGGTGTACGGATCGTAGCGAACTGAGAAGGGCACCGACTCGGTGCCAC
Hs_Pah_1241_peg2_tevo-rv	ATGGTCTCGCGCGCTCAATCCTTTGGGTGTACGGATCGTAGCGAACTGAGAAGCACCGACTCGGTGCCAC
Hs_Pah_1241_peg3_tevo-rv	ATGGTCTCGCGCGCTCAATCCTTTGGGTGTATGGATCGTAGCGAACTGCACCGACTCGGTGCCAC
Hs_Pah_143_peg1_tevo-rv	ATGGTCTCGCGCGAAGAAGTTGGTGCTCTTGCCAAAGTATTGCACCGACTCGGTGCCAC
Hs_Pah_143_peg2_tevo-rv	ATGGTCTCGCGCGAAGAAGTTGGTGCACTCGCCAAAGTATTGCACCGACTCGGTGCCAC
Hs_Pah_143_peg3_tevo-rv	ATGGTCTCGCGCGAAGAAGTTGGTGCACTTGCGAAAGTATTGCACCGACTCGGTGCCAC
Hs_Pah_194_peg1_tevo-rv	ATGGTCTCGCGCGAAGGTCTAGATTCAATATGCGTCAGGTTTACGCACCGACTCGGTGCCAC
Hs_Pah_194_peg2_tevo-rv	ATGGTCTCGCGCGAAGGTCTAGATTCAATATGCGTCAGGTTTACATGCACCGACTCGGTGCCAC
Hs_Pah_194_peg3_tevo-rv	ATGGTCTCGCGCGAGGTCTAGATTCAATATGCGTCAGGTTTACATGCACCGACTCGGTGCCAC
Hs_Pah_754_peg1_tevo-rv	ATGGTCTCGCGCGCACCCAAGAAATCCCTACTACTAAGCAGGCCAGCACCGACTCGGTGCCAC
Hs_Pah_754_peg2_tevo-rv	ATGGTCTCGCGCGCACCCAAGAAATCCCGACTACTAAGCAGGCCAGCACCGACTCGGTGCCAC
Hs_Pah_754_peg3_tevo-rv	ATGGTCTCGCGCGCCCAAGAAATCCCGACTCGAAAGCAGCACCGACTCGGTGCCAC
PKU_CHANGE_1_fw	CTTTCCCTACACGACGCTCTTCCGATCTGGTTTCCCATTTCCCATCATCATT
PKU _CHANGE_1_rv	GGAGTTCAGACGTGTGCTCTTCCGATCTCTGGGAAGTGTGTACATGTATGG
PKU _CHANGE_2_fw	CTTTCCCTACACGACGCTCTTCCGATCTAGCATGTATGGGTTCCGAGG
PKU _CHANGE_2_rv	GGAGTTCAGACGTGTGCTCTTCCGATCTGAAACCAGTTTCAGCACGGTC
PKU _CHANGE_3_fw	CTTTCCCTACACGACGCTCTTCCGATCTATTAGGGAGGAGGGTAGAAGTGT
PKU _CHANGE_3_rv	GGAGTTCAGACGTGTGCTCTTCCGATCTGCAGTACATGAGCTTCCGC
PKU _CHANGE_4_fw	CTTTCCCTACACGACGCTCTTCCGATCTCAGGGCAAGCAGGTAGATGT
PKU _CHANGE_4_rv	GGAGTTCAGACGTGTGCTCTTCCGATCTACCTGTCGCCCTTCAGTTTG
PKU _CHANGE_5_fw	CTTTCCCTACACGACGCTCTTCCGATCTAGTATACCACTGTTTGTGCATTCC
PKU _CHANGE_5_rv	GGAGTTCAGACGTGTGCTCTTCCGATCTGCAAGTACGCTGCACACAAT
HTS_DNMT1_fw	CTTTCCCTACACGACGCTCTTCCGATCTNNNNNNGTCTTCCCCCACTCTCTTGC
HTS_DNMT1_rv	GGAGTTCAGACGTGTGCTCTTCCGATCTNNNNNNCCCCCAATATATGCCTCGGC
peg-DNMT1_spacer_fw	ATGGTCTCGCACCGCGGGCTGGAGCTGTTCGCGCGTTTCAGAGCTATGCTGGAAACAGC
peg-DNMT1_tevo_rv	ATGGTCTCGCGCGCTGGAGCTGTTCGCGCTGCCATCTTGCACCGACTCGGTGCCAC
DNMT1_CHANGE_1_fw	CTTTCCCTACACGACGCTCTTCCGATCTCTCAGCCGGACGCCCAATTA
DNMT1_CHANGE_1_rv	GGAGTTCAGACGTGTGCTCTTCCGATCTCCCGCAGCAGCCCTGT
DNMT1_CHANGE_2_fw	CTTTCCCTACACGACGCTCTTCCGATCTACAGGATGTGATATCGGAGGC
DNMT1_CHANGE_2_rv	GGAGTTCAGACGTGTGCTCTTCCGATCTCATGTGATCCACACACGCTT
DNMT1_CHANGE_3_fw	CTTTCCCTACACGACGCTCTTCCGATCTCGAAGGGGAAAACCCAGGAA
DNMT1_CHANGE_3_rv	GGAGTTCAGACGTGTGCTCTTCCGATCTGGTGGCACTGCTAGATCTCC

DNMT1_CHANGE_4_fw	CTTTCCCTACACGACGCTCTTCCGATCTAGCAACTTGAAAAGACTGTGCC
DNMT1_CHANGE_4_rv	GGAGTTCAGACGTGTGCTCTTCCGATCTAGGAGCTACGCAGAACCCTTC
DNMT1_CHANGE_5_fw	CTTTCCCTACACGACGCTCTTCCGATCTTCTCTTTTTGAATCTCCATAGCCCA
DNMT1_CHANGE_5_rv	GGAGTTCAGACGTGTGCTCTTCCGATCTTCACTTGTGAATGTAACACGGC

Supplementary Table 3 | Oligonucleotides used for RT-qPCR.

SYBR_RT_Fw	GAAGGGCTGCAACACAACTG
SYBR_RT_Rev	AGCCCAGATTACCTCGGTCT
SYBR_Cas_Fw	ACCATCGACCGGAAGAGGTA
SYBR_Cas_Rev	TCGCTTGTTCCTGGTGTCTC
SYBR_mRPLP0_FW	TGAGATTCGGGATATGCTGTTGG
SYBR_mRPLP0_Rev	CGGGTCCTAGACCAGTGTTCT

Supplementary Table 4 | chemically modified pegRNAs used in this study.

**Synthetic pegRNA PKU-SM (3'MP protected)**	mG*mC*mC*_spacer_scaffold_UUCCGAGUCUUUCACUGCACACAGUmA^mC^A
**Synthetic DNA-mod pegRNA PKU-SM (3'MP protected)**	mG*mC*mC*_spacer_scaffold_dTdTdCdCdGdAdGdTdCdTdTdTdCdAdCdTdGCACACAGUmA^mC^A
**Synthetic pegRNA PKU-SM (3'MS protected)**	mG*mC*mC*_spacer_scaffold_UUCCGAGUCUUUCACUGCACACAGU*mA*mC*mA
**Synthetic pegRNA DNMT1 (3'MP protected)**	mC*mG*mG*_spacer_scaffold_AAGAUGGCAGCGCGAACAGCUCmC^mA^G
**Synthetic DNA-mod pegRNA DNMT1 (3'MP protected)**	mC*mG*mG*_spacer_scaffold_dAdAdGdAdTdGdGdCdAdGdCdGCGAACAGCUCmC^mA^G
**Synthetic pegRNA DNMT1 (3'MS protected)**	mC*mG*mG*_spacer_scaffold_AAGAUGGCAGCGCGAACAGCUC*mC*mA*mG
**Synthetic La accessible pegRNA DNMT1**	mC*mG*mG*_spacer_scaffold_AAGAUGGCAGCGCGAACAGCUCCAGUU*mU*mU*mUU
**Synthetic La accessible pegRNA PKU**	mG*mC*mC*_spacer_scaffold_UUCCGAGUCUUUCACUGCACACAGUACAUUU*mU*mU*mUU

**Modification Description**	**Notation when used in sequence**
**2'O-methyl (2OMe)**	mA, mC, mG, mU
**2'-ribo 3'-phosphorothioate (S)**	A*, C*, G*, U*
**Deoxy (D)**	dA, dC, dG, dT
**2'-ribo 3'-phosphonoacetate (PACE)**	**A^, C^, G^, U^**
