# Supplementary material for: Treatment of a metabolic liver disease in mice with a transient prime editing approach
Source: Nat Biomed Eng. 2025 May 20;9(10):1705–18. doi: 10.1038/s41551-025-01399-4 (PMC12532708; doi:10.1038/s41551-025-01399-4)
Supplement: Supplementary file 2 — Reporting Summary [file 41551_2025_1399_MOESM2_ESM.pdf]

nature portfolio

Corresponding author(s): Gerald Schwank - NBME-24-0139C

Last updated by author(s): May 5, 2025

Reporting Summary

Nature Portfolio wishes to improve the reproducibility of the work that we publish. This form provides structure for consistency and transparency in reporting. For further information on Nature Portfolio policies, see our Editorial Policies and the Editorial Policy Checklist.

Statistics

For all statistical analyses, confirm that the following items are present in the figure legend, table legend, main text, or Methods section.

n/a	Confirmed	
☐	☒	The exact sample size (*n*) for each experimental group/condition, given as a discrete number and unit of measurement
☐	☒	A statement on whether measurements were taken from distinct samples or whether the same sample was measured repeatedly
☐	☒	The statistical test(s) used AND whether they are one- or two-sided<br>*Only common tests should be described solely by name; describe more complex techniques in the Methods section.*
☐	☒	A description of all covariates tested
☒	☐	A description of any assumptions or corrections, such as tests of normality and adjustment for multiple comparisons
☐	☒	A full description of the statistical parameters including central tendency (e.g. means) or other basic estimates (e.g. regression coefficient) AND variation (e.g. standard deviation) or associated estimates of uncertainty (e.g. confidence intervals)
☐	☒	For null hypothesis testing, the test statistic (e.g. *F*, *t*, *r*) with confidence intervals, effect sizes, degrees of freedom and *P* value noted<br>*Give P values as exact values whenever suitable.*
☒	☐	For Bayesian analysis, information on the choice of priors and Markov chain Monte Carlo settings
☒	☐	For hierarchical and complex designs, identification of the appropriate level for tests and full reporting of outcomes
☐	☒	Estimates of effect sizes (e.g. Cohen's *d*, Pearson's *r*), indicating how they were calculated

Our web collection on statistics for biologists contains articles on many of the points above.

Software and code

Policy information about availability of computer code

Data collection	Next-generation sequencing (NGS) data was collected and demultiplexed by Illumina NovaSeq Control software (v1.7) and MiSeq Control software (v3.1 and v4.0). Images were taken using Zeiss software Zen2.
Data analysis	Data analysis and visualization was performed using Python 3.9 and 3.10. Cutadapt (3.1) was used to trim sequencing reads. For characterization of endogenous editing and unintended edits, CRISPResso2 (2.2.7) was used. For statistical analysis, SciPy (1.6.3) and Prism (9.0.0) was used. Images were analyzed by Fiji ImageJ software (v1.51n).

For manuscripts utilizing custom algorithms or software that are central to the research but not yet described in published literature, software must be made available to editors and reviewers. We strongly encourage code deposition in a community repository (e.g. GitHub). See the Nature Portfolio guidelines for submitting code & software for further information.

Data

Policy information about availability of data

All manuscripts must include a data availability statement. This statement should provide the following information, where applicable:

- Accession codes, unique identifiers, or web links for publicly available datasets
- A description of any restrictions on data availability
- For clinical datasets or third party data, please ensure that the statement adheres to our policy

Measured editing rates used for the analysis and figures in this study are provided as Supplementary files. Illumina sequencing data are available in the Sequence Read Archive (SRA) under the accession number PRJNA947564 and PRJNA1258720.

Research involving human participants, their data, or biological material

Policy information about studies with human participants or human data. See also policy information about sex, gender (identity/presentation), and sexual orientation and race, ethnicity and racism.

Reporting on sex and gender	not applicable to this study
Reporting on race, ethnicity, or other socially relevant groupings	not applicable to this study
Population characteristics	not applicable to this study
Recruitment	not applicable to this study
Ethics oversight	not applicable to this study

Note that full information on the approval of the study protocol must also be provided in the manuscript.

Field-specific reporting

Please select the one below that is the best fit for your research. If you are not sure, read the appropriate sections before making your selection.

☒ Life sciences ☐ Behavioural & social sciences ☐ Ecological, evolutionary & environmental sciences

For a reference copy of the document with all sections, see nature.com/documents/nr-reporting-summary-flat.pdf

Life sciences study design

All studies must disclose on these points even when the disclosure is negative.

Sample size	No statistical analysis was used to predetermine the sample size. Sample sizes for in vivo experiments were determined based on literature precedence for genome editing experiments (Villiger et al. 2018, 10.1038/s41591-018-0209-1). Sample size for in vitro experiments were determined based on previous studies in the field which generated reproducible results with similar setups (Villiger et al. 2021, 10.1016/j.omtn.2021.08.025).
Data exclusions	No animals and no data were excluded.
Replication	Experiments were performed in three independent replicates, performed on different days, unless otherwise noted. All attempts of replication were successful.
Randomization	For in vitro experiments, no samples were randomized since the ID of the samples was necessary for downstream analysis. The allocation of the cell populations for experiments was done at random. For in vivo experiments, the mice were assigned to the treated or untreated groups by litters.
Blinding	Experiments were rationally designed and executed. Researchers were not blinded to group allocation. The assays performed in this study do not rely on subjective evaluation, as they generate objective and quantitative data.

Reporting for specific materials, systems and methods

We require information from authors about some types of materials, experimental systems and methods used in many studies. Here, indicate whether each material, system or method listed is relevant to your study. If you are not sure if a list item applies to your research, read the appropriate section before selecting a response.

Materials & experimental systems

n/a	Involved in the study
☐	☒ Antibodies
☐	☒ Eukaryotic cell lines
☒	☐ Palaeontology and archaeology
☐	☒ Animals and other organisms
☒	☐ Clinical data
☒	☐ Dual use research of concern
☒	☐ Plants

Methods

n/a	Involved in the study
☒	☐ ChIP-seq
☒	☐ Flow cytometry
☒	☐ MRI-based neuroimaging

Antibodies

Antibodies used	1. mouse anti-Cas9 (1:1'000; Cat. No. #14697T; Cell Signaling)<br>2. rabbit anti-GAPDH (1:10'000; Cat. No. ab181602; abcam)<br><br>Secondary:<br>IRDye 800CW Goat anti-Rabbit IgG Secondary Antibody (Cat. No. 926-32211, LI-COR bio, 1:15'000)<br>IRDye 680RD Goat anti-Mouse IgG Secondary Antibody (Cat. No. 926-68070, LI-COR bio, 1:15'000)
Validation	Validation statements by the manufacturers:<br><br>1. mouse anti-Cas9 (1:1'000; Cat. No. #14697T; Cell Signaling). Monoclonal antibody is produced by immunizing animals with recombinant protein specific to the amino terminus of Cas9 from Streptococcus pyogenes. Cas9 (S. pyogenes) (7A9-3A3) Mouse mAb recognizes transfected levels of total Cas9 protein. This antibody does not cross-react with Cas9 (S. aureus), FnCpf1, and AsCpf1 proteins. Application: This antibody has been validated for Western Blotting, Immunofluoresence and flow cytometry.<br><br>2. rabbit anti-GAPDH (1:10'000; Cat. No. ab181602; abcam) Anti-GAPDH antibody [EPR16891] ab181602 is a rabbit monoclonal antibody. Suitable for human, mouse and rat samples. Specificity and sensitivity confirmed in IHC with multi-tissue microarray (TMA) validation. Application: It is validated for use in GAPDH western blotting, IHC, immunofluorescence and flow cytometry.

Eukaryotic cell lines

Policy information about cell lines and Sex and Gender in Research

Cell line source(s)	HEK293T (ATCC CRL-3216), K562 (ATCC CCL-243)
Authentication	Cells were authenticated by the supplier by STR analysis.
Mycoplasma contamination	All cells were initially tested for mycoplasma by the supplier. Cells were also periodically tested during experimentation. No mycoplasma contamination was found.
Commonly misidentified lines<br>(See ICLAC register)	No commonly misidentified cell lines were used.

Animals and other research organisms

Policy information about studies involving animals; ARRIVE guidelines recommended for reporting animal research, and Sex and Gender in Research

Laboratory animals	Only animals of the species Mus musculus were used. Mice strains were C57BL/6J (Strain #:000664) and Pahenu2 (Strain #:029218) mice. All mice were littermates. Mice were housed in a pathogen-free animal facility at the Institute of Pharmacology and Toxicology of the University of Zurich. Mice were kept in a temperature- and humidity-controlled room (21°C, 50% RH) on a 12-hour light-dark cycle. Mice were fed a standard laboratory chow (Kliba Nafag no. 3437 with 18.5% crude protein) and genotyped at weaning. Mice were injected with AAVs and / or LNPs via the tail vein from 6 to 12 weeks of age.
Wild animals	No wild animals were used in this study.
Reporting on sex	Similar amount of animals from both sexes were used. No sex-specific experiments were carried out.
Field-collected samples	No field collected samples were used in this study.
Ethics oversight	Mouse experiments were performed in accordance with protocols approved by the Kantonales Veterinäramt Zürich.

Note that full information on the approval of the study protocol must also be provided in the manuscript.

Plants

Seed stocks	No plants were used in this study.
Novel plant genotypes	No plants were used in this study.
Authentication	No plants were used in this study.
